# Coagulation Risk Prediction in Patients With Liver Failure: Integrated Meta-Analysis and Machine Learning Model Study

**DOI:** 10.2196/76348

**Published:** 2025-12-08

**Authors:** Hao Wang, Tao He, Liang Ren, Tingjun Zhang

**Affiliations:** 1 Affiliated Hospital of North Sichuan Medical College Nanchong China

**Keywords:** artificial liver support system, liver failure, coagulation function, meta-analysis, machine learning, predictive modeling

## Abstract

**Background:**

Liver failure often results in significant coagulation dysfunction, which is a major complication. Artificial liver support systems (ALSS) have been used to ameliorate coagulation parameters, but the dynamic nature of these improvements and the development of predictive models remain insufficiently explored.

**Objective:**

This study aimed to evaluate the effects of ALSS on coagulation function and to develop a dynamic prediction model using machine learning techniques to predict the improvement trends of coagulation parameters.

**Methods:**

A systematic search was conducted in PubMed, Embase, and other databases to identify relevant studies, resulting in 18 studies comprising 1771 patients. A meta-analysis was performed to assess the impact of ALSS on coagulation parameters, including international normalized ratio (INR), prothrombin time (PT), activated partial thromboplastin time (APTT), and fibrinogen levels. In addition, clinical data from the Medical Information Mart for Intensive Care database were used to construct prediction models using logistic regression, extreme gradient boosting, random forest, and long short-term memory networks.

**Results:**

Meta-analysis results showed that ALSS significantly improved INR, PT, APTT, and fibrinogen levels (all *P*<.05), with the treatment efficacy varying by modality. Among the machine learning models, the random forest model demonstrated the best performance, achieving an area under the curve of 92.12%. Dynamic INR was identified as the key predictor for coagulation abnormalities.

**Conclusions:**

This study systematically evaluated the effects of ALSS on coagulation function in patients with liver failure, demonstrating significant improvements in key parameters such as INR, PT, and APTT, with efficacy varying across different treatment modalities. Simultaneously, a machine learning model built using intensive care unit clinical data exhibited strong predictive capability for identifying the risk of coagulation dysfunction, particularly useful in supporting early-stage clinical recognition of high-risk patients and guiding personalized coagulation management strategies. It is important to emphasize that this model is positioned as a dynamic risk alert and assessment tool, intended to assist clinical baseline evaluation and nursing interventions, rather than serving as direct validation of ALSS therapeutic efficacy.

## Introduction

Liver failure represents a critical clinical condition frequently complicated by severe coagulopathy, which significantly increases bleeding risk [1,2]. The disruption of coagulation homeostasis may lead to life-threatening complications, including gastrointestinal hemorrhage and intracranial bleeding, while potentially triggering disseminated intravascular coagulation that exacerbates multiorgan dysfunction [3,4]. As a crucial therapeutic intervention, the artificial liver support system (ALSS) demonstrates the potential for improving coagulation parameters through its detoxification, synthetic, and metabolic substitution functions [5-8]. However, the mechanisms underlying ALSS-mediated coagulation improvement remain insufficiently investigated, particularly regarding differential effects among various modalities (eg, plasma exchange [PE] and molecular adsorbent recirculating system [MARS]). Clinical decision-making is further challenged by the lack of reliable dynamic monitoring indicators and predictive tools for optimal treatment timing and duration adjustment [9,10]. Furthermore, the highly individualized nature of coagulation dynamics in patients with liver failure cannot be adequately captured by conventional static tests (eg, prothrombin time [PT] and activated partial thromboplastin time [APTT]) [11-13]. Therefore, a comprehensive investigation of ALSS-induced dynamic coagulation changes and the development of accurate predictive models are clinically imperative for optimizing individualized treatment strategies. Addressing this scientific challenge will directly impact bleeding risk management and overall prognosis in patients with liver failure.

Although several studies have investigated the effects of ALSS on coagulation function in patients with liver failure, most suffer from limitations, such as small sample sizes and single-center designs, which compromise the reliability and generalizability of their findings [14]. More notably, the majority of these studies focus solely on short-term outcomes (eg, changes within 24 hours posttreatment), failing to capture the long-term dynamic evolution of coagulation parameters [15]. Methodologically, conventional statistical analyses (eg, *t* tests and ANOVA) are inadequate for characterizing temporal trends in coagulation indicators, limiting their utility for developing precise predictive models [16-18]. While meta-analyses can synthesize evidence from multiple studies to enhance statistical power [19], no study to date has systematically evaluated the differential effects of ALSS modalities on coagulation function. Advances in machine learning have demonstrated significant potential for medical data mining and predictive modeling [20,21]; however, their application in monitoring coagulation dysfunction in liver failure remains nascent. A critical gap persists in integrating multisource heterogeneous clinical data (eg, electronic health records, laboratory results, and vital signs), as existing studies have yet to leverage the full potential of large-scale public databases like Medical Information Mart for Intensive Care (MIMIC). These methodological limitations hinder clinicians and nurses from accurately predicting and promptly intervening in coagulation function changes among patients with liver failure.

This study uses a multidimensional and multimethod research strategy to achieve 3 key objectives. First, we conduct a systematic review and meta-analysis to comprehensively evaluate the effects of ALSS on coagulation parameters (eg, international normalized ratio [INR], PT, APTT, and fibrinogen) in patients with liver failure, while comparing the efficacy of different treatment modalities. Second, leveraging large-scale clinical data from the MIMIC database, we apply advanced machine learning algorithms to develop a dynamic, time-series predictive model for assessing coagulation function. Third, using interpretability techniques (eg, Shapley Additive Explanations [SHAP] value analysis), we identify key predictive factors influencing coagulation dynamics, providing a theoretical foundation for clinical interventions. From a scientific perspective, this study is the first to elucidate the dynamic mechanisms by which ALSS modulates coagulation function in liver failure, deepening our understanding of coagulopathy in this context and advancing precision medicine in the field. Clinically, our findings offer three major practical applications: (1) an objective, data-driven decision-support tool to optimize individualized ALSS treatment strategies; (2) early detection of coagulation abnormalities, enabling timely interventions to reduce bleeding complications; and (3) improved allocation of nursing resources, enhancing health care efficiency and ultimately patient outcomes. These innovations will significantly elevate the standard of care for patients with liver failure and serve as a methodological reference for research on other organ support therapies.

This study adopts a dual-track integrative strategy, combining meta-analysis with machine learning modeling, aiming to comprehensively elucidate the interventional value of ALSS on coagulation function in patients with liver failure and to uncover the underlying mechanisms of coagulation risk prediction across multiple levels. The meta-analysis, based on 18 clinical studies, systematically evaluated the short-term and long-term improvement effects of different ALSS modalities—including PE, MARS, and double plasma perfusion (PP)—on key coagulation indicators, such as INR, PT, APTT, and fibrinogen. The results confirmed the clinical benefits of ALSS in reducing the risk of coagulation dysfunction and provided an evidence-based foundation for subsequent predictive modeling. However, due to the limited number of patients who received ALSS treatment in the MIMIC database, the training population for the machine learning model focused on a broader cohort of patients with liver failure. The goal was to construct a generalizable and efficient dynamic coagulation risk assessment tool applicable to real-time bedside alerts and optimized resource allocation. We consider this model a “baseline tool” for risk stratification and timing judgment before ALSS initiation in clinical practice, laying a methodological foundation for the development of specialized models based on larger, ALSS-specific datasets in the future. Therefore, this study achieves an organic integration from high-level evidence synthesis to individualized risk prediction, not only enhancing the understanding of the clinical benefits of ALSS but also providing a practical path for the precise implementation of personalized interventional strategies in liver failure management.

Accordingly, the primary aim of this study is to systematically evaluate the short-term and long-term effects of ALSS on coagulation function in patients with liver failure and to further construct a clinically valuable dynamic risk prediction model for coagulation dysfunction. The specific objectives are as follows: (1) to quantify, through systematic review and meta-analysis, the improvement effects of different ALSS modalities (such as PE, MARS, and PP) on major coagulation parameters (INR, PT, APTT, and fibrinogen); (2) to use real-world clinical data from the MIMIC database and apply multiple machine learning algorithms to develop predictive models for early identification of coagulation dysfunction within the next 24 hours; and (3) to integrate model interpretability techniques (eg, SHAP analysis) to identify key predictive variables associated with changes in coagulation function, thereby providing theoretical support for personalized treatment and nursing interventions. Through this integrated strategy, the study seeks to promote the advancement of precision ALSS therapy and offers a novel pathway for bleeding risk management and prognosis improvement in patients with liver failure.

## Methods

### Literature Search and Screening

This meta-analysis and systematic review were conducted in accordance with the PRISMA (Preferred Reporting Items for Systematic Reviews and Meta-Analyses) guidelines [22]. A comprehensive literature search was performed across multiple databases, including PubMed, Embase, Web of Science, and the Cochrane Library, using a combination of Medical Subject Headings terms and free-text keywords to ensure thoroughness and precision. The search strategy incorporated key terms such as liver failure, artificial liver support (ALS), blood coagulation, and hemostasis, optimized with Boolean operators (AND and OR) to balance sensitivity and specificity (refer to Table S1 in Multimedia Appendix 1 for detailed search queries). To account for variations in database syntax, the search strategy was tailored to each platform. In addition, manual screening of reference lists from included studies was performed to identify potentially eligible articles not captured by the initial database search. To confirm the novelty of this study, a search for registered systematic reviews was conducted in the PROSPERO database.

The screening process was independently carried out by 2 researchers (HW and TH) using EndNote (Clarivate) for reference management. The selection procedure consisted of two phases: (1) initial screening based on titles and abstracts to exclude clearly irrelevant studies, followed by (2) full-text review of potentially eligible articles, with documented reasons for exclusion. Any discrepancies between reviewers were resolved through discussion or adjudication by a third researcher (LR) to ensure objectivity. Interrater agreement was assessed using Cohen κ coefficient to quantify screening consistency. To enhance transparency, the study selection process was visualized using a PRISMA flow diagram, providing a clear and reproducible overview of the screening stages.

### Inclusion and Exclusion Criteria

Inclusion criteria consisted of (1) study design: randomized controlled trials, cohort studies, or case-control studies; (2) participants: patients diagnosed with liver failure according to international or national guidelines (eg, Asian Pacific Association for the Study of the Liver, European Association for the Study of the Liver, and American Association for the Study of Liver Diseases); (3) intervention: use of ALSS, including but not limited to PE, double PE, MARS, or continuous blood purification, either alone or in combination with standard therapies (eg, medications or supportive care); (4) outcome reporting: studies must report coagulation parameters at multiple time points; and (5) key outcomes: at least one of the following coagulation-related indicators: PT, APTT, D-dimer, INR, or fibrinogen.

Exclusion criteria included (1) publication type: reviews, conference abstracts, book chapters, or case reports without original data suitable for meta-analysis; (2) language: non-English or non-Chinese publications; and (3) data limitations: studies that did not report changes in coagulation function before and after treatment, reported only single time-point measurements, or provided incomplete or nonextractable data.

### Risk of Bias Assessment

This study used the Newcastle-Ottawa scale (NOS) to systematically evaluate the risk of bias in the included studies, ensuring the reliability and scientific validity of the meta-analysis results. The NOS assesses study quality across three domains with a maximum score of 9 points: (1) selection (maximum 4 points)—evaluates whether cases and controls were clearly defined, representative of the target population, and selected using an appropriate study design; (2) comparability (maximum 2 points)—assesses whether key confounding factors were accounted for through matching or statistical adjustment; and (3) outcome or exposure assessment (maximum 3 points)—examines the reliability of outcome or exposure measurement, adequacy of follow-up duration, and handling of attrition. Based on the NOS scores, study quality was categorized as follows: ≥7 points=high quality (low risk of bias); 5-6 points=moderate quality (moderate risk of bias); ≤4 points=low quality (high risk of bias).

Two independent reviewers (HW and TH) conducted bias assessments, with cross-verification of results. Discrepancies were resolved through discussion or adjudication by a third reviewer (LR). The risk of bias was visualized using a risk of bias graph, and a detailed summary was provided in Multimedia Appendix 1. All assessments and graphical representations were generated using Review Manager (RevMan 5.4 [Cochrane Collaboration]).

### Data Extraction

The following data were extracted from each included study: (1) study characteristics: first author, publication year, study design (randomized controlled trial, cohort study, or case-control study), country, and journal; (2) participant characteristics: patient type (viral hepatitis-related liver failure), sample size, sex ratio, mean age, baseline disease severity (Child-Pugh score and model for end-stage liver disease score), and coagulation function (PT, APTT, D-dimer, INR, and fibrinogen); (3) methodological features: type of ALS therapy (PE, hemoperfusion, MARS, continuous renal replacement therapy [CRRT]) and treatment protocol (single vs multiple sessions, treatment duration); and (4) outcome measures: the primary outcomes included changes in coagulation parameters (PT, APTT, INR, and fibrinogen before and after treatment). If a study reported multiple follow-up measurements, the longest follow-up data were extracted to assess long-term effects. For missing data, the original authors were contacted for clarification. If unavailable, multiple imputations were applied. Two researchers (LR and TJ) independently extracted all data, with cross-verification to resolve discrepancies; a third researcher (HW) adjudicated unresolved disagreements. The final dataset was entered into Microsoft Excel and double-checked to ensure accuracy and consistency.

### Meta-Analysis Statistical Methods

This study used the “meta” package in R software (version 4.2; R Core Team) to conduct a meta-analysis evaluating the dynamic changes in coagulation function during ALS therapy in patients with liver failure. Heterogeneity across studies was assessed using the *I*^2^ statistic and Q-test. A fixed-effects model was applied when *I*^2^<50% and *P*>.05, indicating negligible heterogeneity. In cases of significant heterogeneity (*I*^2^≥50% and *P*≤.05), a random-effects model was used, followed by an exploration of potential heterogeneity sources. The pooled effect size was calculated based on data type: standardized mean difference or mean difference with 95% CIs for continuous variables, and odds ratios or risk ratios with 95% CIs for dichotomous variables. Results were visualized using forest plots to illustrate individual and pooled effect estimates.

To further investigate the key factors influencing coagulation function, we conducted subgroup analyses based on the following variables: type of ALS (PE vs hemoperfusion vs MARS vs CRRT), liver failure classification (acute liver failure [ALF], acute-on-chronic liver failure [ACLF], and chronic liver failure), and etiology of liver failure (viral hepatitis-induced vs cirrhosis-induced liver failure).

To assess the robustness of the findings, a sensitivity analysis was performed using the leave-one-out method, in which each study was systematically excluded before rerunning the meta-analysis. This approach allowed us to evaluate whether any single study disproportionately influenced the overall results. A significant change in the pooled effect size following the exclusion of a particular study would suggest that it was a key driver of the meta-analytic findings.

Publication bias was preliminarily assessed using funnel plots and further examined through Egger linear regression test and Begg test. A statistically significant result (Egger test *P*<.05) was considered indicative of potential publication bias.

### Data Extraction and Cleaning From Public Databases

This study used the MIMIC-IV-3.1 database, released by the Massachusetts Institute of Technology (MIT), which contains clinical data from patients admitted to the intensive care unit (ICU) at Beth Israel Deaconess Medical Center (BIDMC) between 2008 and 2019. Data extraction was performed using Navicat Premium (v15.0.12; PremiumSoft CyberTech Ltd) and structured query language. The inclusion criteria were (1) first-time ICU admission, (2) ICU stay duration exceeding 48 hours, and (3) a diagnosis of liver failure based on *International Classification of Diseases* codes (K704, K7040, K7041, K72, K720, K7200, K7201, K721, K7210, K7211, K729, K7290, K7291, and K9182).

The extracted clinical variables comprised 3 categories: demographic and admission characteristics, vital signs, and laboratory results. Demographic data included age, sex, race, admission time, ICU admission and discharge time, and hospital ID. Vital signs, such as heart rate, respiratory rate, oxygen saturation, body temperature, systolic blood pressure (BP), and diastolic BP, were recorded based on the first measurement after ICU admission. Laboratory tests covered liver and kidney function (alanine aminotransferase, aspartate aminotransferase, total bilirubin [TBIL], creatinine, urea, and albumin), complete blood count (white blood cell count, platelet count, hemoglobin, and hematocrit), inflammatory markers (C-reactive protein), and coagulation parameters, including the INR (PT), partial thromboplastin time (PTT), and functional fibrinogen. All coagulation-related measurements during the ICU stay were extracted for dynamic predictive modeling, whereas static features were derived from the first recorded value after ICU admission. Data cleaning and imputation followed established protocols [23].

The clinical data used in this study were derived from the publicly available MIMIC-IV database (version 4.0), jointly developed by MIT and BIDMC. The database has been approved by the Institutional Review Board of BIDMC and the ethics committee of MIT and is accessible to qualified researchers who have completed the required credentialing course. All authors of this study completed the Collaborative Institutional Training Initiative program data usage training and obtained PhysioNet Credentialed Access to MIMIC. This study strictly adhered to the data usage agreement and did not involve any personally identifiable patient information, thus complying with ethical standards and requiring no additional ethical approval.

### Data Cleaning and Missing Value Handling

Before model construction, we conducted systematic cleaning and preprocessing of the raw clinical data from the MIMIC-IV v3.1 database. All data were merged based on the unique patient identifier (subject_id), integrating demographic information, vital signs, laboratory test results, and ICU admission and discharge times across multiple tables. The analysis window was limited to the first 24 hours following ICU admission.

To ensure the stability and interpretability of variables in the model, only candidate features with high sampling frequency and clear clinical relevance were included, and variables with a missing rate greater than 20% were excluded. For features with a missing rate between 5% and 20%, multiple imputation was performed to preserve the covariate structure as much as possible. For features with sporadic missing values, group-level median or mean imputation was applied. Categorical variables such as sex and race, which had low rates of missingness, were imputed using the mode.

For dynamic time-series variables such as INR and PTT, we applied intrasample linear interpolation or forward and backward filling methods to handle missing data. In addition, the number of missing values per variable was included as an auxiliary input feature in the model to reflect potential uncertainty in the data. To eliminate the influence of extreme outliers, we set clinically reasonable cutoff thresholds based on existing literature and expert knowledge, applying percentile truncation or direct exclusion as appropriate.

All numerical variables were standardized using *z* score normalization before model input to eliminate differences in measurement scale, while categorical variables were converted into dummy variables using one-hot encoding.

### Sliding Window Feature Extraction

For each coagulation function record, the collection time (charttime) was treated as the current time point. We then extracted data from the preceding 24-hour window to derive statistical features for 3 key coagulation indicators: INR, PTT, and functional fibrinogen. For each indicator, 6 statistical measures were computed: mean, maximum (max), minimum (min), standard deviation (SD), the most recent value (last), and the slope of the linear regression trend (slope). This process generated a total of 18 time-series dynamic features. To ensure that the extracted features accurately reflected dynamic changes during ICU stays, only valid in-ICU records were included, thereby constructing an ICU-specific dynamic prediction dataset.

### Label Construction

This study adopts a 24-hour observation window following the current time point to construct a binary label variable. The label is determined based on clinically established diagnostic thresholds for coagulopathy. A patient is classified as having a “coagulopathy event” (label=1) if any of the following abnormal indicators occur within the observation window: INR>1.5, PTT>60 seconds, or fibrinogen<150 mg/dL. Conversely, if none of these criteria are met, the case is labeled as 0. Records with insufficient laboratory data to determine abnormality are assigned a missing label (label=NaN) and excluded before modeling.

### Static Model Construction

The static model incorporates patient features recorded upon ICU admission, including demographic characteristics (eg, sex, age, and race), liver and kidney function metrics, vital signs, and statistical features (mean, maximum, minimum, SD, last recorded value, and slope) of coagulation parameters (INR, PTT, and fibrinogen). The target variable is defined as the occurrence of a coagulation abnormality within 24 hours of the current time point, where an abnormality is identified if any of the following criteria are met: INR>1.5, PTT>60 seconds, or fibrinogen<150 mg/dL. Three supervised learning algorithms—logistic regression (LR), random forest (RF), and extreme gradient boosting (XGBoost)—were used for model development.

To prevent data leakage, the dataset was partitioned into training and testing sets (80:20 ratio) using a group-based hold-out split, ensuring that samples from the same patient (subject ID) were exclusively allocated to either set. During model training, stratified group k-fold cross-validation (5 folds, preserving class balance and group separation) was applied to evaluate performance metrics, including the mean area under the curve (AUC), *F*_1_-score, accuracy, sensitivity, and specificity. Subsequently, the final models were refit on the entire training set and assessed for generalizability on the independent test set.

To prevent information leakage caused by the presence of the same patient in both the training and test sets, we adopted a group-based splitting strategy based on subject ID for static model development. Patients were randomly assigned to the training and test sets in an 80:20 ratio, resulting in 2764 patients (80% of the total 3456) in the training set and 692 patients (20%) in the test set. On the training set, we developed several static models—including LR, RF, and XGBoost—and evaluated their performance using the StratifiedGroupKFold method (5-fold cross-validation with stratification and grouping). This approach ensured robust estimation of average performance metrics, including AUC, *F*_1_-score, accuracy, sensitivity, and specificity. Model-specific parameters are detailed in Table S2 in Multimedia Appendix 1. Based on this, final models were refitted on the entire training set and evaluated on the independent test set to assess their generalizability.

### Dynamic Long Short-Term Memory Model Construction

For dynamic modeling, we used a long short-term memory (LSTM) recurrent neural network to analyze the constructed time-series data. Each sample consisted of a sequence of 5 consecutive time points, with each time point represented by sliding-window statistical features. The label indicated whether coagulation dysfunction occurred within 24 hours after the last time point in the sequence. The specific parameters are provided in Table S2 in Multimedia Appendix 1.

### Model Evaluation and Visualization

The performance of each model was evaluated on the test set using the following metrics: accuracy, *F*_1_-score, AUC, sensitivity, specificity, and precision. Visual representations included the receiver operating characteristic (ROC) curve, precision-recall (PR) curve, calibration curve, decision curve analysis, and a confusion matrix heatmap. The AUC values were computed with 95% CIs using the bootstrap method.

### Robustness Validation and Model Interpretation

To enhance model robustness, the static model was further evaluated using 5-fold StratifiedGroupKFold cross-validation. This approach ensured data stratification by subject_id, maintaining label distribution while preventing data leakage. Performance metrics were reported as mean (SD).

Subsequently, model interpretability analysis was conducted using the best-performing RF model. Global feature importance rankings were derived using the Gini index, while SHAP values quantified the average contribution of each variable to model predictions. The most critical clinical predictors were identified and visualized to illustrate their impact on model outputs.

### Ethical Considerations

This study does not contain any studies with human participants or animals performed by any of the authors.

## Results

### Literature Search Results

**Figure 1:**
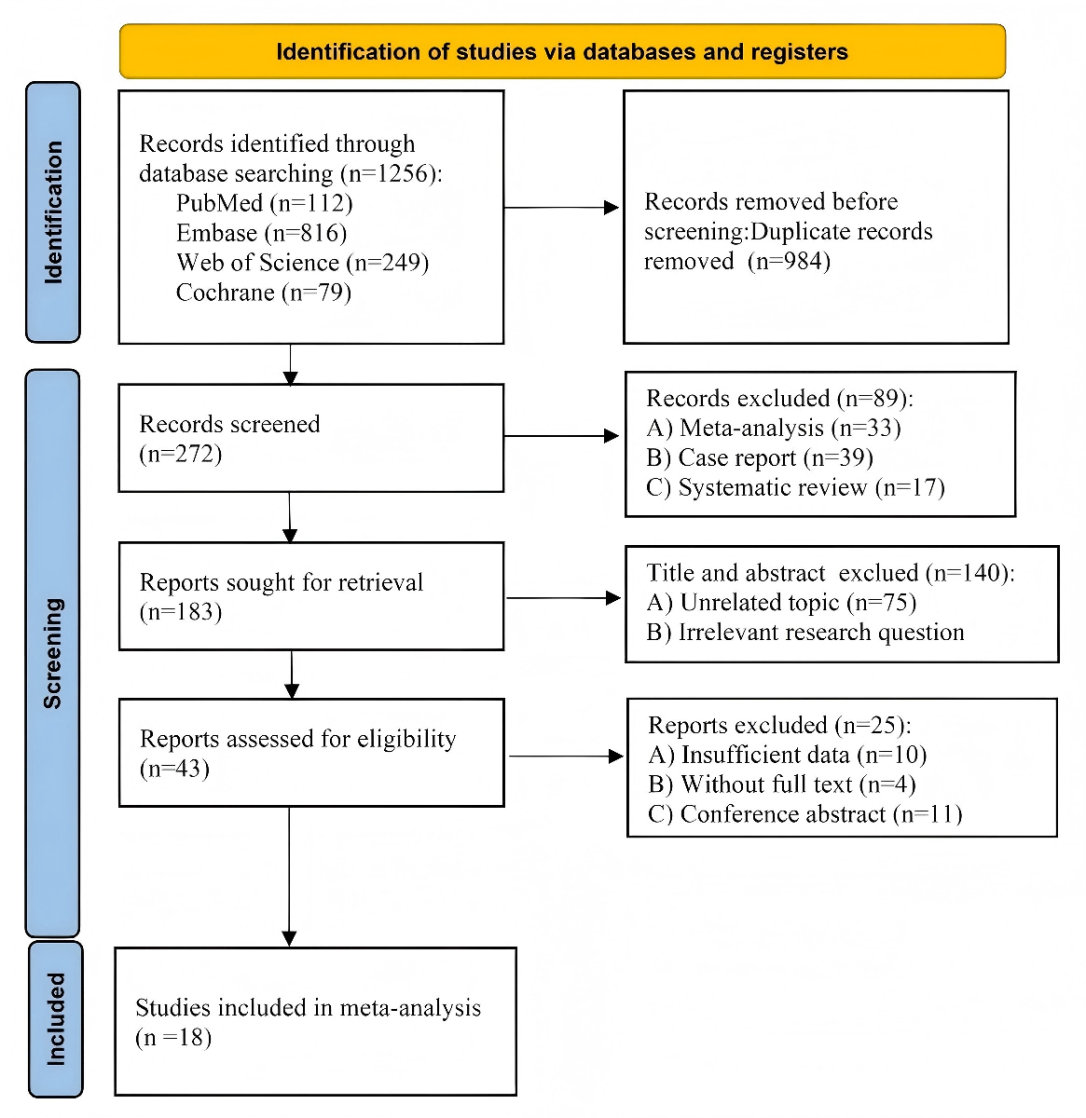
PRISMA (Preferred Reporting Items for Systematic Reviews and Meta-Analyses) literature screening flowchart.

As illustrated in Figure 1, a total of 1256 studies were retrieved from the database. After removing 263 duplicate records, 993 studies underwent title and abstract screening. Following initial screening, 921 studies that did not meet the inclusion criteria were excluded, leaving 72 studies for full-text evaluation. After a rigorous assessment, an additional 59 studies were excluded due to mismatches in study design, intervention methods, or outcome measures, resulting in 18 studies being included in the systematic review and meta-analysis.

All 18 included studies were cohort or case-control designs, examining various ALSS (eg, PE, MARS, and PP). These studies collectively involved 1771 participants and reported changes in coagulation parameters—including INR, PT, APTT, and fibrinogen—before and after treatment. The study populations comprised patients with ALF and ACLF, with etiologies such as viral hepatitis, toxic exposure, infections, and metabolic disorders. The key characteristics of the included studies are summarized in Table 1.

**Table 1:** Basic features of included studies.

Study	Research design	Participant category	Etiology	Age (y)	Sex ratio (male/female), mean (SD)	Baseline disease severity	Treatment type	Indicator
Kounis et al [24]	Cohort study	ACLF^a^	Cirrhosis	21	50.6 (7.8)	MELD^b^≥30	PE^c^+CRRT^d^	a
Ninan et al [25]	Cohort study	ALF^e^	Toxication	36	31.67 (13.56)	MELD≥30	PE	bc
Yang et al [26]	Cohort study	ALF	Infection	39	7 (6.67)	Child-Pugh Grade C	PE+CRRT	a
Hu et al [27]	Cohort study	ALF	Toxication	21	1.83 (0.89)	Child-Pugh Grade C	PE	bc
Huang et al [28]	Cohort study	ACLF	HBV^f^	365	46.52 (11.38)	20≤MELD<30	PE+PP^g^	abd
Lee et al [29]	Cohort study	ACLF	Liver transplantation	15	49.7 (7.5)	—^h^	MARS^i^	a
Li et al [30]	Case-control study	ACLF	HBV	45	50.4 (10.7)	20≤MELD<30	PE	ab
Mohanka et al [31]	Cohort study	ALF	Toxication	19	32.0 (9.6)	20≤MELD<30	PE+CVVHDF^j^	a
Sorodoc et al [32]	Case-control study	ALF	Toxication	6	37.13 (18.37)	CTP^k^ Grade A	MARS	b
Chen et al [33]	Cohort study	ACLF	Virus	250	—	20≤MELD<30	PE	bd
Falkensteiner et al [34]	Cohort study	ALF	Primary liver failure	49	59 (13.71)	MELD≥30	MARS	d
Colak and Ocak [35]	Cohort study	ALF	Infection	24	52 (18.25)	10≤PRISM^l^<20	PE+CVVHDF	ad
Gong et al [36]	Cohort study	ALF	—	5	60.2 (6.25)	—	SAE^m^	a
Kulkarni et al [37]	Cohort study	ALF	HBV	42	23.5 (3.625)	MELD≥30	PE	ab
Stöckert et al [38]	Cohort study	ALF	Toxication	43	52 (18.4)	MELD<20	PE	a
Pawaria et al [39]	Cohort study	ALF	Metabolic diseases	37	9 (7.41)	NWI≥11	PE	a
Hung et al [40]	Case-control study	ACLF	HBV	20	50.6 (2.86)	—	PE	c
Wan et al [41]	Cohort study	ACLF	HBV	60	50.7 (9.2)	20≤MELD<30	PE	a

^a^ACLF: acute-on-chronic liver failure.

^b^MELD: model for end-stage liver disease.

^c^PE: plasma exchange.

^d^CRRT: continuous renal replacement therapy.

^e^ALF: acute liver failure.

^f^HBV: hepatitis B virus.

^g^PP: plasma perfusion.

^h^Not available.

^i^MARS: molecular adsorbent recirculating system.

^j^CVVHDF: continuous veno-venous hemodiafiltration.

^k^CTP: Child–Turcotte–Pugh.

^l^PRISM: pediatric risk of mortality.

^m^SAE: severe adverse event.

### Risk of Bias Assessment

All 18 observational studies included in this meta-analysis were evaluated for risk of bias using the NOS. Among them, 16 studies were rated as high quality (NOS score≥7), indicating robust methodological rigor in case selection, comparability between groups, and exposure assessment. The remaining 2 studies were classified as moderate quality (score=6), primarily due to incomplete follow-up data or unclear exposure assessment methods. No studies were deemed low quality (score≤5).

Overall, the included studies demonstrated high methodological quality, enhancing the reliability of our meta-analysis findings. Detailed NOS scores are presented in Table 2.

**Table 2:** Risk of bias assessment summary: quality evaluation for each included study using the modified Newcastle-Ottawa scale.

Study		Selection^a^					Comparability^a^		Exposure^a^				Total		Interpretation	
		Q1	Q2	Q3	Q4	Q5		Q6		Q7	Q8					
**Case-control studies**																
	Sorodoc et al [32]	1	1	1	1	2		1		1	—^b^	8		High quality		
	Hung et al [40]	1	1	1	1	2		1		1	1	9		High quality		
**Cross-sectional studies**																
	Kounis et al [24]	1	1	1	1	2		1		1	1	9		High quality		
	Ninan et al [25]	1	1	1	1	2		1		1	1	9		High quality		
	Yang et al [26]	1	—	1	1	2		1		1	1	8		High quality		
	Hu et al [27]	1	1	1	1	2		1		—	—	7		High quality		
	Huang et al [28]	1	—	1	1	2		1		1	1	8		High quality		
	Lee et al [29]	1	1	1	1	2		1		1	1	9		High quality		
	Mohanka et al [31]	1	—	1	1	2		1		1	1	8		High quality		
	Chen et al [33]	1	1	1	1	1		1		—	—	6		Moderate quality		
	Falkensteiner et al [34]	1	1	1	1	2		1		1	—	8		High quality		
	Colak and Ocak [35]	1	1	1	1	2		1		—	—	7		High quality		
	Gong et al [36]	1	1	1	1	1		1		—	—	6		Moderate quality		
	Kulkarni et al [37]	1	1	1	1	2		1		1	1	9		High quality		
	Stöckert et al [38]	1	—	1	1	2		1		1	1	8		High quality		
	Pawaria et al [39]	1	1	1	1	2		1		1	1	9		High quality		
	Wan et al [41]	1	—	1	1	2		1		1	1	8		High quality		
	Li et al [30]	1	1	1	1	1		1		—	—	6		High quality		

^a^The scoring system ranges from 0 to 9 points. Higher scores indicate better methodological quality.

^b^Not available.

### Meta-Analysis Results: INR

**Figure 2:**
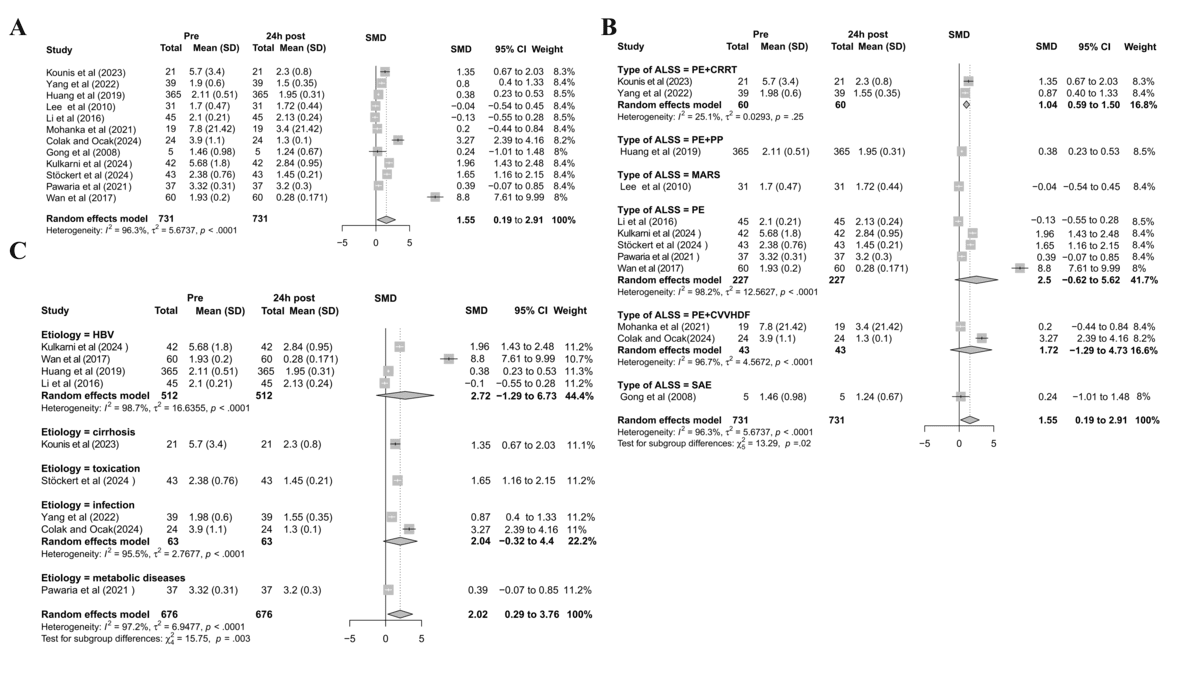
Meta-analysis of the short-term effect of artificial liver support (ALS) on international normalized ratio improvement and its heterogeneity sources. (A) Forest plot comparing international normalized ratio changes within 24 hours before and after ALS treatment; (B) subgroup analysis stratified by ALS modality; (C) subgroup analysis stratified by etiology. ALSS: artificial liver support system; CRRT: continuous renal replacement therapy; CVVHDF: continuous veno-venous hemodiafiltration; HBV: Hepatitis B virus; MARS: molecular adsorbent recirculating system; PE: plasma exchange; PP: plasma perfusion; SAE: severe adverse event; SMD: standardized mean difference [33,35,37-40,44-48,50].

A total of 12 studies (encompassing 731 patients) were included to evaluate changes in INR before and 24 hours after ALS treatment. The meta-analysis revealed a significant reduction in INR posttreatment, although with considerable heterogeneity (Figure 2A).

To investigate potential sources of heterogeneity, we conducted 3 subgroup analyses. In the ALS modality subgroup analysis, different treatment modalities exhibited significant variations in INR improvement. PE alone demonstrated the most pronounced reduction in INR, albeit with high heterogeneity. In contrast, the PE+CRRT group showed stable and significant effects alongside markedly reduced heterogeneity (Figure 2B), suggesting that treatment modality may be a key factor influencing efficacy and heterogeneity. The etiology-based subgroup analysis indicated that patients with hepatitis B virus (HBV) or infections exhibited more significant therapeutic effects, whereas those with cirrhosis or metabolic diseases showed smaller or nonsignificant effect sizes. Intergroup differences were statistically significant (Figure 2C), implying that underlying etiology may contribute to variations in INR improvement. The disease-type subgroup analysis revealed that patients with ACLF had greater INR improvement than patients with ALF, although the difference was not statistically significant (Figure S1A in Multimedia Appendix 1).

Sensitivity analysis (Figure S1B in Multimedia Appendix 1) demonstrated consistent pooled effect directions and minimal fluctuations in *I*^2^ upon sequential exclusion of individual studies, supporting the robustness of the findings. In addition, the funnel plot exhibited approximate symmetry (Figure S1C in Multimedia Appendix 1), indicating a low likelihood of publication bias and further strengthening the reliability of the conclusions.

In summary, this study demonstrates that ALS significantly improves INR levels in patients with liver failure, with treatment efficacy potentially influenced by therapeutic modality and underlying etiology.

**Figure 3:**
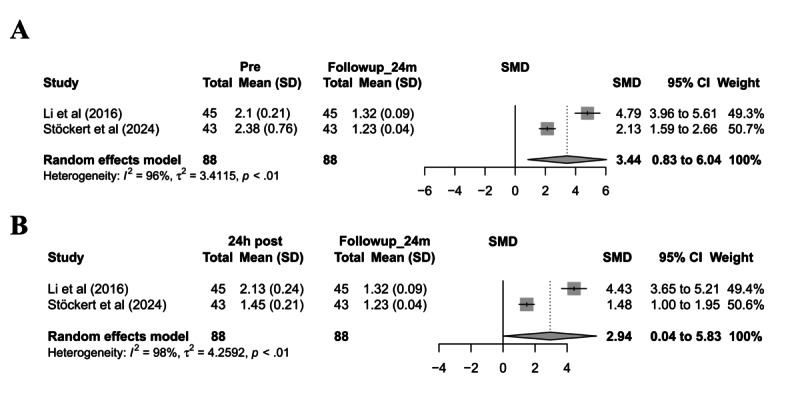
Meta-analysis of the effects of artificial liver support (ALS) on international normalized ratio (INR) improvement at different posttreatment time points. (A) Forest plot comparing INR changes between pre-ALS and 24 months post-ALS; (B) forest plot comparing INR changes between 24 hours and 24 months post-ALS. SMD: standardized mean difference [39,47].

To evaluate long-term coagulation function changes following ALS therapy, we conducted a meta-analysis of 2 studies reporting INR values before treatment and at 24 months posttreatment. The results demonstrated a statistically significant reduction in INR at 24 months compared with baseline, although with considerable heterogeneity (Figure 3A). Furthermore, we compared INR changes between the 24-hour and 24-month time points to assess differences in short-term versus long-term efficacy. The pooled analysis, which included the same 2 studies, revealed that INR remained significantly lower at 24 months than at 24 hours, with statistical significance, suggesting that ALS therapy may have a long-term effect on improving coagulation function (Figure 3B).

Despite moderate heterogeneity, the findings consistently indicate a progressive decline in INR over long-term follow-up, supporting the stability and durability of ALS therapy in enhancing coagulation.

In conclusion, ALS therapy significantly and persistently improves INR levels in patients with liver failure, although its efficacy may be influenced by treatment modalities and underlying etiologies.

### Meta-Analysis of PT Levels Before and 24 Hours After Treatment

**Figure 4:**
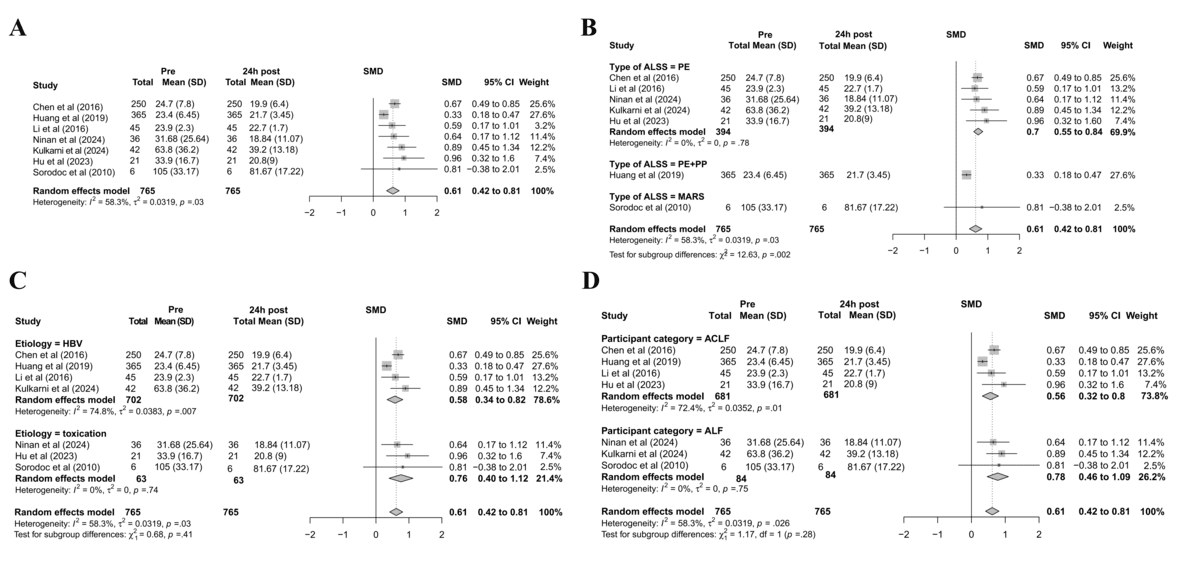
Meta-analysis of PT changes before and after artificial liver support (ALS) treatment, with subgroup analyses. (A) Forest plot of PT changes within 24 hours post-ALS versus pre-ALS; (B) subgroup analysis of PT changes by ALS type; (C) subgroup analysis by etiology; (D) subgroup analysis by disease classification. ACLF: acute-on-chronic liver failure; ALF: acute liver failure; ALSS: artificial liver support system; HBV: Hepatitis B virus; MARS: molecular adsorbent recirculating system; PE: plasma exchange; PP: plasma perfusion; SMD: standardized mean difference [34,36,37,39,41,42,46].

A total of 7 studies (combined sample size=765 patients) were included to evaluate changes in PT 24 hours before and after ALS treatment. The pooled analysis revealed a significant reduction in PT levels posttreatment, with moderate heterogeneity observed (Figure 4A).

Subgroup analysis by ALS type demonstrated statistically significant differences between groups, with the PE subgroup exhibiting no heterogeneity (*I*^2^=0). This suggests that the type of ALS may contribute to heterogeneity and influence the degree of coagulation improvement (Figure 4B). Etiology-based subgroup analysis indicated no significant differences between HBV-related liver failure and toxic liver failure groups, with the latter subgroup showing no heterogeneity (*I*^2^=0). This implies that etiology may be a source of heterogeneity (Figure 4C). Disease-type subgroup analysis found no significant difference between ACLF and ALF groups (*P*=.28), with the ALF subgroup displaying no heterogeneity (*I*^2^=0), suggesting disease type as a potential source of heterogeneity (Figure 4D).

Sensitivity analysis demonstrated that the pooled effect size remained stable after the sequential exclusion of individual studies, with no substantial changes in heterogeneity, indicating robust findings (Figure S2A in Multimedia Appendix 1). Funnel plot symmetry suggested no significant publication bias (Figure S2B in Multimedia Appendix 1).

In conclusion, ALS significantly improves PT levels, with treatment type, etiology, and disease classification identified as potential key sources of heterogeneity.

### Meta-Analysis of Fibrinogen Levels Before and 24 Hours After Treatment

**Figure 5:**
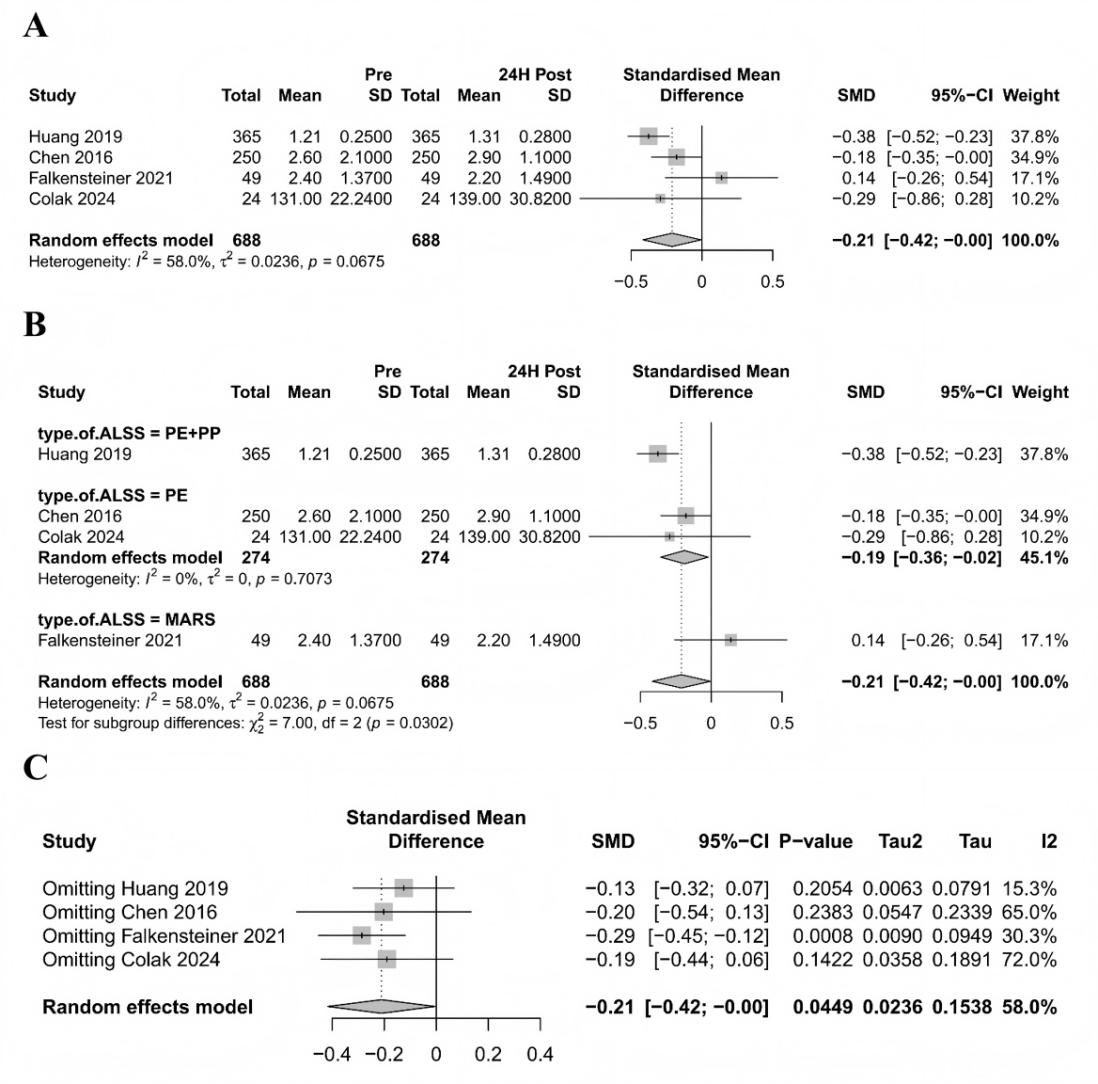
Meta-analysis of the effect of artificial liver support (ALS) on fibrinogen levels in patients with liver failure. (A) Forest plot of fibrinogen level changes pre- and post-ALS; (B) subgroup analysis by ALS type; (C) sensitivity analysis using the leave-one-out method. ALSS: artificial liver support system; MARS: molecular adsorbent recirculating system; PE: plasma exchange; PP: plasma perfusion; SMD: standardized mean difference [37,42-44].

This meta-analysis included 4 studies comprising 688 patients to evaluate changes in fibrinogen levels before and after ALS treatment. The results demonstrated a slight decrease in fibrinogen levels posttreatment, with moderate heterogeneity observed in the overall analysis (Figure 5A).

Subgroup analysis by ALS therapy type revealed that the PE+PP subgroup exhibited the most significant reduction in fibrinogen levels, with statistically significant intergroup differences. Notably, the PE subgroup showed no heterogeneity (*I*^2^=0%) (Figure 5B), suggesting that treatment type may be a key factor influencing fibrinogen level changes and a potential source of heterogeneity. In contrast, subgroup analyses based on etiology and disease type showed no statistically significant intergroup differences, nor did they substantially alter heterogeneity (Figure S3A-B in Multimedia Appendix 1).

Sensitivity analysis indicated that the exclusion of individual studies only significantly reduced heterogeneity (to 15.3%) upon the removal of Huang et al [28] (Figure 5C), identifying it as a potential source of heterogeneity. Funnel plot analysis demonstrated a generally symmetrical study distribution with no evident publication bias (Figure S3C in Multimedia Appendix 1).

In conclusion, ALS therapy has a limited effect on fibrinogen levels, and the observed heterogeneity may primarily stem from differences in treatment modalities.

### Meta-Analysis of APTT Levels Before and 24 Hours After Treatment

**Figure 6:**
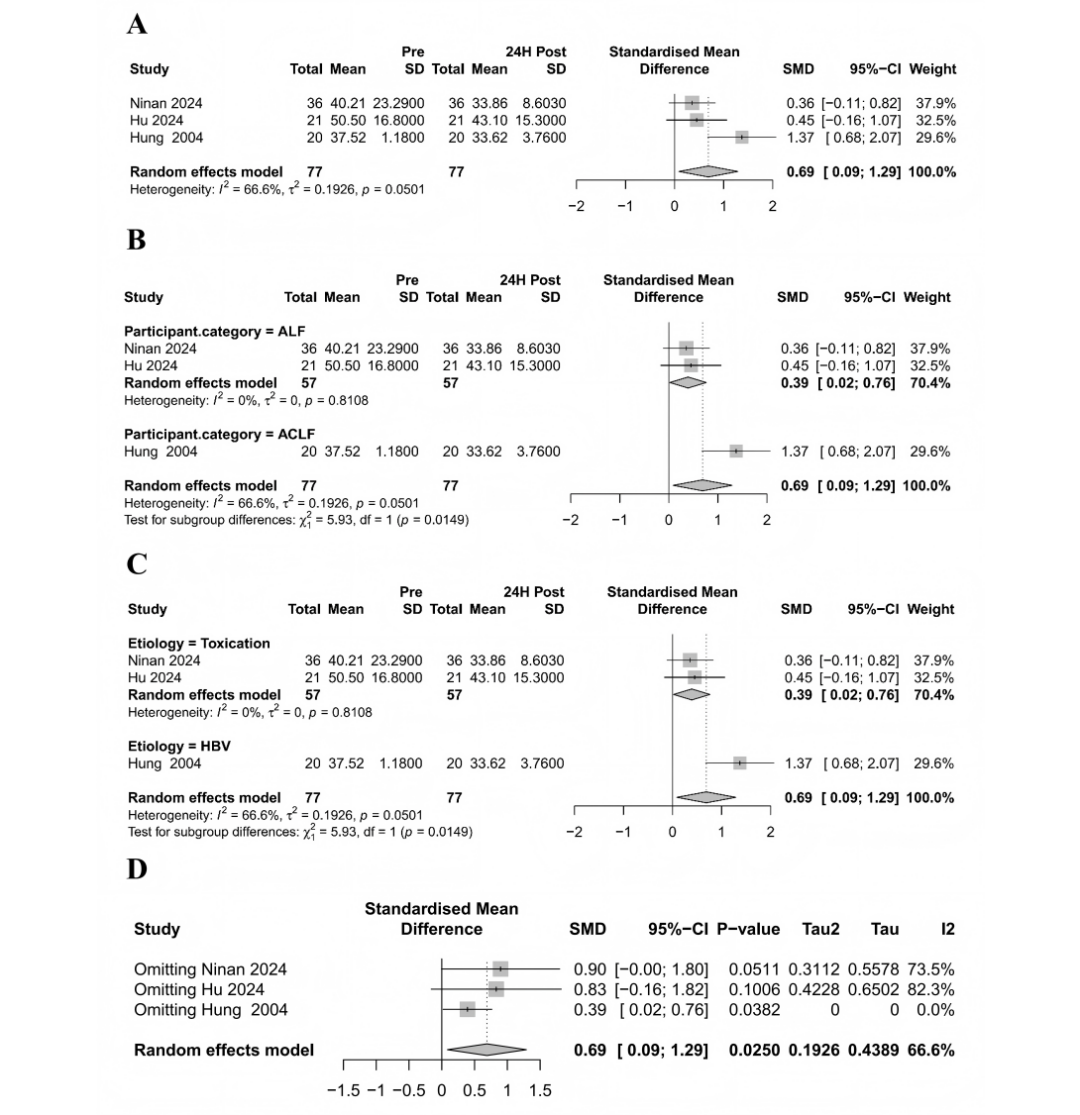
Meta-analysis of the effect of artificial liver support on APTT in patients with liver failure. (A) Forest plot of APTT changes pre- and post–artificial liver support; (B) subgroup analysis by disease type; (C) subgroup analysis by etiology; (D) sensitivity analysis. ACLF: acute-on-chronic liver failure; ALF: acute liver failure; ALSS: artificial liver support system; HBV: Hepatitis B Virus; SMD: standardized mean difference [34,36,49].

A total of 3 studies (comprising 77 patients) were included to evaluate changes in APTT before and after ALS therapy. The meta-analysis revealed a significant reduction in APTT levels posttreatment, though moderate heterogeneity was observed (Figure 6A).

To investigate potential sources of heterogeneity, subgroup analyses were performed based on disease type and etiology. The disease-type subgroup analysis demonstrated that patients with ACLF exhibited a greater improvement in APTT than patients with ALF, with a statistically significant intergroup difference. Notably, heterogeneity within the ALF subgroup was 0%, suggesting that disease type may contribute to heterogeneity and influence the degree of coagulation improvement (Figure 6B). The etiology-based subgroup analysis indicated that patients with HBV showed a more pronounced APTT improvement than those with toxic liver injury, with significant intergroup differences and 0% heterogeneity in the toxic etiology subgroup. This further supports etiology as a potential source of heterogeneity and a modifier of coagulation function (Figure 6C).

Sensitivity analysis confirmed the robustness of the pooled effect size after excluding Hung et al [40], with heterogeneity (*I*^2^) decreasing to 0% (Figure 6D). Funnel plot symmetry suggested no significant publication bias (Figure S4 in Multimedia Appendix 1).

In summary, ALS therapy significantly improves APTT levels, with heterogeneity likely attributable to differences in disease type and etiology.

This study demonstrates that ALS therapy significantly enhances multiple coagulation parameters in patients with liver failure, particularly in reducing INR and PT while improving APTT. The long-term improvement in INR suggests long-term therapeutic benefits in coagulation support. Treatment efficacy varies significantly depending on the artificial liver modality (eg, PE, double PE, and MARS), etiology (eg, viral hepatitis vs toxic liver failure), and disease type (ALF vs ACLF), which are key contributors to interstudy heterogeneity. In contrast, improvements in fibrinogen levels were relatively limited, with variability primarily influenced by treatment modality. These findings underscore the importance of individualized coagulation management strategies in ALS therapy, optimizing treatment efficacy while providing evidence-based guidance for coagulation monitoring and clinical care.

### Dynamic Coagulation Risk Prediction in Patients With Liver Failure

Based on previous meta-analysis findings, this study confirms that ALS therapy significantly improves coagulation function in patients with liver failure across multiple time points, demonstrating clear dynamic trends in core indicators (INR, PT, APTT, and fibrinogen). However, current clinical practice still lacks individualized predictive tools for coagulation dysfunction, hindering precise intervention and dynamic monitoring. To address this gap, we further integrated clinical data from the MIMIC database to develop a machine learning–based predictive model. By leveraging dynamic coagulation indicators as key features, this model explores predictive pathways for coagulation abnormalities, aiming to provide an intelligent decision-support tool for the early identification of high-risk patients.

A total of 3456 ICU patients with liver failure were included from the MIMIC-IV database based on strict inclusion and exclusion criteria. Patients were stratified by ID and randomly assigned to the training cohort (n=2764) and independent validation cohort (n=692). Table 3 presents the baseline demographic characteristics, vital signs, and laboratory parameters of all patients. Overall, most features were well-balanced between the training and validation sets, with no statistically significant differences. Specifically, there was no significant difference in age distribution between the 2 cohorts (*P*=.57), with 2262 patients (65.5%) younger than 65 years. A statistically significant difference was observed in sex distribution (*P*<.001), with a slightly higher proportion of males (2140/3456, 60.9%). Regarding race, White patients accounted for the largest proportion (1942/3456, 56.2%), while the proportion of Asian patients was slightly higher in the validation cohort (*P*=.02). For laboratory parameters—including liver function markers (alanine aminotransferase, aspartate aminotransferase, TBIL, and alkaline phosphatase), renal function (creatinine and urea), inflammatory marker (C-reactive protein), complete blood count (platelet count, hemoglobin, and hematocrit), coagulation indicators (INR_PT, PTT, and fibrinogen), and vital signs (heart rate, respiratory rate, oxygen saturation, and BP)—the overall distributions between the training and validation sets were consistent. Only hemoglobin (*P*=.02) and hematocrit (*P*=.02) showed mild differences, suggesting slight variations in hemoglobin levels among some patients.

In conclusion, the training and validation cohorts exhibited a strong balance in key clinical and laboratory characteristics, supporting their appropriateness for subsequent model development and validation.

**Table 3:** Baseline characteristics of patients in training and validation cohorts: demographic data, vital signs, laboratory parameters, and comparative statistics.

Group			All data		Train data		Test data		*P* value
N			3456		2764		692		nan^a^
**Age (y), n (%)**									
	≥65	1194 (34.6)		948 (34.3)		246 (35.6)		.57	
	<65	2262 (65.5)		1816 (65.7)		446 (64.6)		.57	
**Sex, n (%)**									
	Female	1352 (39.1)		1124 (40.7)		228 (33)		<.001	
	Male	2104 (60.9)		1640 (59.3)		464 (67)		<.001	
**Race**									
	Black	274 (7.9)		210 (7.6)		64 (9.2)		.17	
	White	1942 (56.2)		1568 (56.7)		374 (54)		.22	
	Asian	100 (2.9)		70 (2.5)		30 (4.3)		.02	
	Other	1140 (33)		916(33.1)		224 (32.4)		.73	
ALT^b^, mean (SD)			446.69 (950.08)		435.07 (926.86)		493.02 (1037.01)		.20
AST^c^, mean (SD)			562.95 (1031.00)		567.76 (1042.23)		543.15 (984.00)		.59
TBIL^d^, mean (SD)			6.20 (8.71)		6.09 (8.62)		6.66 (9.05)		.13
ALP^e^, mean (SD)			145.63 (146.90)		145.82 (152.40)		144.91 (122.78)		.87
Albumin, mean (SD)			2.92 (0.65)		2.92 (0.65)		2.91 (0.67)		.78
PLT^f^, mean (SD)			154.11 (98.77)		153.59 (98.02)		156.27 (101.90)		.56
HGB^g^, mean (SD)			9.99 (2.58)		9.93 (2.57)		10.20 (2.61)		.02
HCT^h^, mean (SD)			30.74 (7.92)		30.57 (7.88)		31.39 (8.06)		.02
CRP^i^, mean (SD)			96.36 (74.60)		96.52 (73.93)		95.77 (77.51)		.94
CRE^j^, mean (SD)			1.94 (1.59)		1.92 (1.58)		1.99 (1.65)		.36
Urea, mean (SD)			37.92 (28.50)		37.97 (28.75)		37.74 (27.55)		.85
HR^k^, mean (SD)			95.01 (21.89)		95.35 (22.25)		93.64 (20.36)		.05
RR^l^, mean (SD)			20.98 (6.62)		20.96 (6.62)		21.07 (6.63)		.72
SpO_2_^m^, mean (SD)			96.65 (21.49)		96.69 (23.94)		96.49 (4.18)		.68
SBP^n^, mean (SD)			115.24 (24.09)		115.37 (24.20)		114.72 (23.64)		.53
DBP^o^, mean (SD)			68.65 (18.43)		68.68 (18.60)		68.54 (17.71)		.85
Fibrinogen, functional, mean (SD)			272.37 (152.93)		269.88 (149.78)		282.29 (164.66)		.11
INR_PT^p^, mean (SD)			1.91 (0.66)		1.92 (0.66)		1.88 (0.64)		.13
PTT^q^, mean (SD)			44.07 (12.49)		44.12 (12.42)		43.88 (12.76)		.66

^a^nan: not a number.

^b^ALT: alanine aminotransferase.

^c^AST: aspartate aminotransferase.

^d^TBIL: total bilirubin.

^e^ALP: alkaline phosphatase.

^f^PLT: platelet count.

^g^HGB: hemoglobin.

^h^HCT: hematocrit.

^i^CRP: C-reactive protein.

^j^CRE: creatinine.

^k^HR: heart rate.

^l^RR: respiratory rate.

^m^SpO_2_: oxygen saturation.

^n^SBP: systolic blood pressure.

^o^DBP: diastolic blood pressure.

^p^INR_PT: international normalized ratio of prothrombin time.

^q^PTT: partial thromboplastin time.

### Model Discriminatory Power and Performance Evaluation

**Figure 7:**
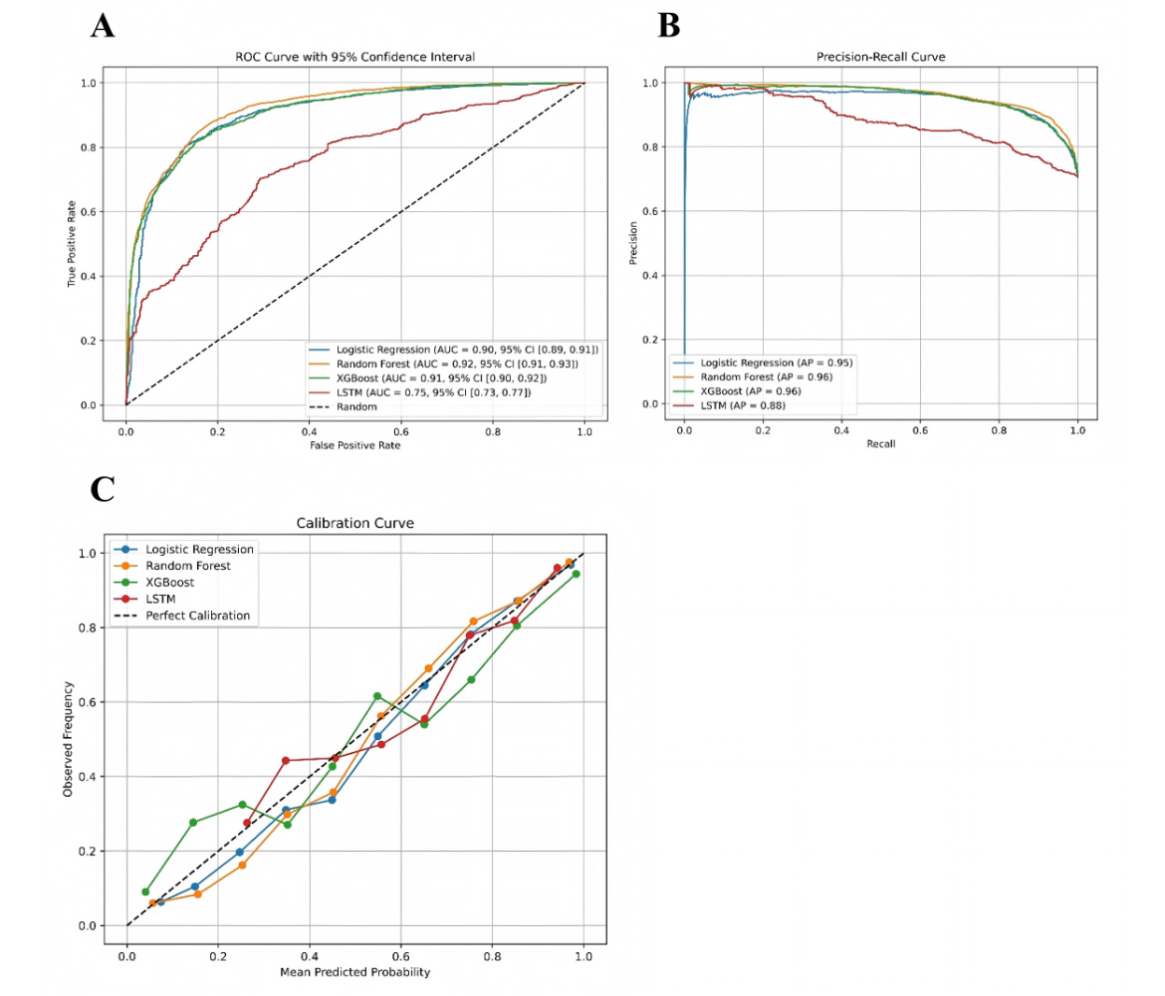
Performance evaluation of machine learning models in predicting coagulation dysfunction in patients with liver failure. (A) Receiver operating characteristic curves comparing the discriminative ability of logistic regression, random forest, extreme gradient boosting, and long short-term memory models in predicting coagulation abnormalities within 24 hours (test set); (B) PR curves assessing model performance in identifying positive cases under class imbalance; (C) calibration curves evaluating the agreement between predicted probabilities and observed event rates. AP: average precision; AUC: area under the curve; LSTM: long short-term memory; XGBoost: extreme gradient boosting; ROC: receiver operating characteristic.

A comprehensive evaluation of model performance was conducted on an independent test set, incorporating metrics such as ROC curves, PR curves, and calibration curves. The ROC curve analysis revealed that the RF model demonstrated the highest discriminatory power, with an AUC of 0.92 (95% CI 0.91-0.93). The XGBoost and LR models exhibited comparable performance, with AUC values of 0.91 (95% CI 0.90-0.92) and 0.90 (95% CI 0.89-0.91), respectively. In contrast, the LSTM dynamic model showed significantly lower discriminative ability, achieving an AUC of only 0.74 (95% CI 0.72-0.76), suggesting limited generalizability under the current data configuration (Figure 7A).

The PR curve analysis further assessed the models’ ability to identify positive cases (ie, predicting coagulation abnormalities within 24 hours). Both RF and XGBoost achieved high average precision (AP) scores of 0.96, maintaining robust precision even at high recall levels. The LR model exhibited a slightly lower AP (0.95) but remained clinically useful. The LSTM model, however, underperformed with an AP of 0.87, reinforcing its limitations in positive predictive accuracy (Figure 7B).

Calibration curve analysis evaluated the agreement between predicted probabilities and observed outcomes. The LR and RF models demonstrated near-perfect calibration across all risk strata, indicating high reliability. The XGBoost model showed stable calibration in moderate-to-high risk ranges but minor deviations in low-risk regions. In contrast, the LSTM model exhibited substantial fluctuations in low-to-moderate risk ranges, reflecting weaker overall calibration (Figure 7C).

The confusion matrix analysis revealed distinct performance advantages among the models in the classification task. The RF model achieved the best classification performance on the test set, while LR and XGBoost exhibited comparable results, both maintaining a high true positive rate with a low misclassification rate. In contrast, although the LSTM model demonstrated superior sensitivity in identifying positive cases, it showed marked limitations in correctly classifying negative samples, resulting in lower specificity and a higher false-positive rate (Figure S5A in Multimedia Appendix 1). Overall, traditional machine learning models outperformed the LSTM in both accuracy and robustness.

To further evaluate the clinical utility of these models, we conducted decision curve analysis to assess their net benefit across different risk thresholds. The results demonstrated that LR, RF, and XGBoost models consistently outperformed the “treat all” and “treat none” strategies across most threshold ranges, indicating strong potential for clinical decision support. Notably, these models provided greater net benefit in intermediate-risk ranges, suggesting their predictive outcomes could reliably guide individualized interventions (Figure S5B in Multimedia Appendix 1).

In summary, static machine learning models—particularly RF—not only exhibited strong predictive discrimination but also demonstrated better calibration performance than the time-series–based LSTM dynamic model. These findings suggest that static models are more suitable for clinical early-warning applications in predicting coagulation dysfunction risk in patients in ICU with liver failure.

Table 4 summarizes the common performance metrics across all models. Overall, the results demonstrate that the RF model achieves the most robust performance, with an AUC of 92.1%, an *F*_1_-score of 91%, an accuracy of 86.9%, a recall (sensitivity) of 93.3%, a precision of 88.8%, and a specificity of 71.7%.

Based on these findings, we select the RF model as our final risk prediction model.

**Table 4:** Performance comparison of 4 machine learning algorithms for dynamic coagulation risk prediction.

Model	*F*_1_-score (%)	Accuracy (%)	Recall (%)	AUC^a^ (%)	Sensitivity (%)	Specificity (%)
LR^b^	89.7	85.1	92.5	90.4	92.5	67.3
RF^c^	91	86.9	93.3	92.1	93.3	71.7
XGBoost^d^	89.7	85.1	92.2	90.9	92.2	68.1
LSTM^e^	82.6	72.2	93.5	75.4	93.5	21

^a^AUC: area under the curve.

^b^LR: logistic regression.

^c^RF: random forest.

^d^XGBoost: extreme gradient boosting.

^e^LSTM: long short-term memory.

### Feature Importance and Model Interpretability Analysis

**Figure 8:**
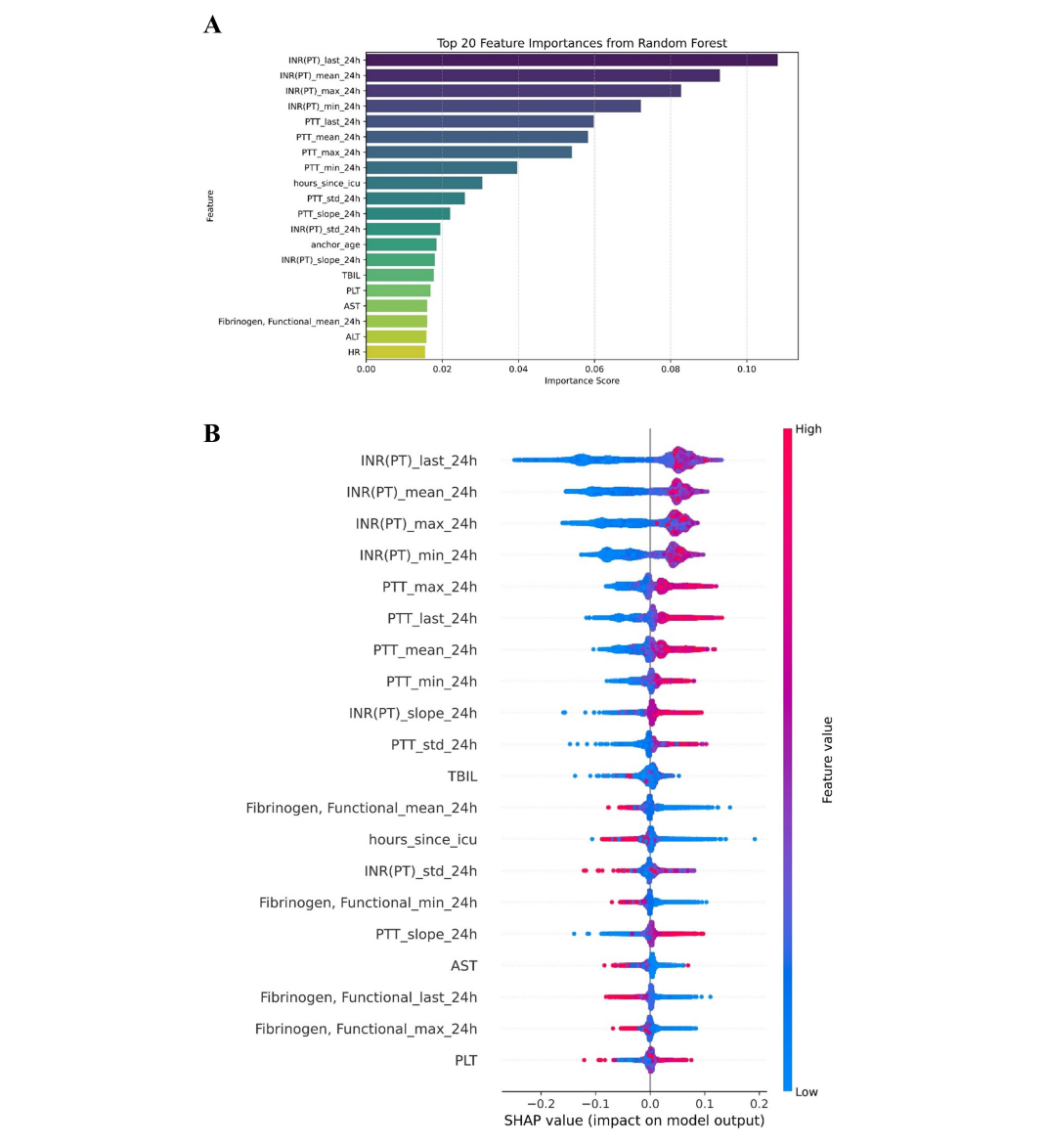
Feature importance and interpretability analysis of the random forest model. (A) Bar plot of feature importance scores; (B) SHapley Additive exPlanations summary plot, where red and blue indicate high and low feature values, respectively, and the x-axis represents the directional impact on predictions. “INR (PT)” (labeled per MIMIC database conventions) is standardized as international normalized ratio in this study; “PTT” refers to activated partial thromboplastin time. ALT: alanine aminotransferase; AST: aspartate aminotransferase; HR: heart rate; INR: international normalized ratio; PLT: platelet count; PT: prothrombin time; PTT: partial thromboplastin time; TBIL: total bilirubin.

To elucidate the decision-making mechanism of the predictive model, this study conducted a feature importance assessment and interpretability analysis based on the top-performing RF model. As illustrated in Figure 8A, among the dynamic features, multiple INR-related indicators—including INR(PT)_last_24h, INR(PT)_mean_24h, INR(PT)_max_24h, and INR(PT)_min_24h—exhibited the highest importance scores, indicating that INR values play a critical role in predicting coagulation abnormalities within the next 24 hours. PTT-related features (eg, PTT_last_24h, PTT_mean_24h, and PTT_max_24h) also ranked among the top 10, further underscoring the significance of dynamic coagulation time in the model’s decision-making process.

To enhance model interpretability, we used SHAP for visual explanation of the RF model. The results demonstrated that elevated INR(PT) and PTT values positively contributed to the prediction of coagulation abnormalities, aligning with established clinical pathophysiological mechanisms. In addition, static variables such as hours_since_icu, fibrinogen, TBIL, and anchor_age exhibited moderate predictive importance (Figure 8B).

In summary, the RF model not only demonstrates superior predictive performance but also maintains strong interpretability, effectively identifying key variables associated with coagulation dysfunction. These findings support early clinical identification of high-risk patients.

## Discussion

### Principal Findings

This study systematically evaluates the dynamic effects of ALS on coagulation function in patients with liver failure by integrating meta-analysis and machine learning modeling based on clinical databases. In addition, it develops an intelligent predictive model for future coagulation abnormalities. The meta-analysis results demonstrate that ALS significantly improves key coagulation parameters, including INR, PT, and APTT, highlighting its critical role in coagulation support for patients with liver failure. These findings align with previous studies [42,43], further reinforcing the evidence-based efficacy of ALS therapy in ameliorating coagulopathy. The maintained reduction in INR during long-term follow-up further underscores the stability and durability of its therapeutic efficacy. Variations in treatment modalities, etiologies, and liver failure subtypes were identified as major sources of heterogeneity in coagulation improvement.

This study adopts a dual-track strategy that integrates high-level evidence synthesis with individualized risk prediction, thereby avoiding the misconception that the meta-analysis and machine learning model are 2 parallel approaches. Specifically, the meta-analysis establishes the overall efficacy and heterogeneity of ALSS in improving coagulation parameters, which provides a rigorous evidence-based rationale for subsequent predictive modeling. Building upon these findings, the machine learning model operationalizes the most clinically relevant indicators—such as INR, PT, and APTT—into a dynamic decision-support tool for real-time risk assessment at the bedside. This methodological pathway transforms population-level evidence into patient-level prediction, thus ensuring both validity and clinical applicability.

Importantly, our results demonstrated that INR was the most significantly improved parameter in the meta-analysis and simultaneously the strongest predictive feature in the machine learning model, underscoring the translational consistency between the 2 components. This alignment exemplifies the synergistic relationship in which meta-analysis informs feature selection and validates the clinical importance of predictors, while the model extends these findings to enable individualized, real-time monitoring. Such integration reflects a broader trend in evidence-based medicine and artificial intelligence, where systematic reviews and meta-analyses provide high-level validity [44], predictive analytics enable dynamic risk assessment [45], and the convergence of these methods enhances clinical decision-making [46,47].

A related study also used MIMIC-IV v3.1 data and applied XGBoost to predict Peripherally Inserted Central Catheter–related thrombosis in patients with sepsis [48]. Compared with that work, our study differs in population (patients with liver failure undergoing ALSS vs patients with sepsis with Peripherally Inserted Central Catheter), target (coagulation abnormalities after ALSS vs catheter-related thrombosis), and methodology (integration of meta-analysis with multiple machine learning models vs single algorithm). Building upon existing literature, this study systematically compares—for the first time—the differential effects of various ALSS (eg, PE, CRRT, and MARS) on coagulation function, thereby addressing a critical research gap and providing new evidence for individualized clinical treatment strategies. These distinctions highlight the novelty and specific contribution of our approach to coagulation risk prediction in the ALSS context. Notably, ALS showed limited efficacy in improving fibrinogen levels, potentially due to differences in fibrinogen clearance efficiency across treatment methods. Subgroup analysis revealed that PE combined with CRRT yielded more consistent coagulation improvements compared with monotherapy, suggesting that hybrid ALS regimens may be more suitable for critically ill patients with severe coagulation dysfunction. In addition, disparities in APTT improvement are primarily influenced by the underlying disease type, underscoring the importance of etiological classification in guiding personalized treatment.

Unlike previous studies that primarily focused on static measurements at single time points [49], this study adopts a dynamic monitoring approach, for the first time, elucidating the evolutionary patterns and predictive value of INR, PT, APTT, and fibrinogen throughout the treatment course. Notably, the dynamic features of INR in the model—including its mean, peak, and trend—demonstrated the highest weighting, confirming its clinical significance as an early warning indicator. This dynamic modeling strategy significantly enhances predictive sensitivity and prospective utility, shifting risk assessment from post hoc evaluation to real-time intervention.

In predictive modeling, this study enhances translational potential by developing a multialgorithm model based on real-world data from MIMIC-IV. Among the tested models, the RF algorithm achieved the highest performance (AUC=92.12%, *F*_1_-score=90.96%) in accurately predicting coagulation abnormalities within 24 hours. Unlike previous studies that primarily relied on single-algorithm approaches such as LR [50], this work systematically compared and integrated multiple machine learning models (including XGBoost and LSTM). Furthermore, SHAP interpretation was used to elucidate feature contributions, improving model transparency and clinical interpretability. The proposed model holds promise as an intelligent clinical decision–support tool in ICU settings, assisting nurses in early identification of high-risk patients for coagulation disorders. By optimizing transfusion strategies, monitoring frequency, and intervention timing, this approach may reduce bleeding-related complications while enhancing the precision and efficiency of coagulation management. Ultimately, this shift from experience-based judgment to data-driven decision-making provides a more reliable foundation for clinical workflows.

In conclusion, ALS significantly improves coagulation parameters (INR, PT, and APTT) in patients with liver failure, while the machine learning model based on dynamic indicators provides accurate risk prediction for coagulation abnormalities. These findings offer valuable support for early intervention and precision management in coagulation care. These results offer strong evidence for implementing earlier and more targeted clinical nursing interventions.

Despite the innovation and clinical utility of this study, several limitations should be acknowledged. First, although this study systematically evaluated the effects of ALSS on coagulation function in patients with liver failure through meta-analysis and developed a dynamic indicator-based coagulation risk prediction model, the constructed predictive model did not specifically target patients who received ALSS treatment. Instead, risk modeling was conducted for the broader population of patients with liver failure. This decision was primarily due to the extremely limited number of ALSS-treated cases identified in the MIMIC-IV database—only 5 patients met the strict inclusion criteria—making the sample size insufficient to meet the basic data requirements for machine learning model development or to ensure model robustness and generalizability. As a compromise, this study focused on the overall liver failure population to explore trends in coagulation function and dynamic risk prediction. This approach provides a foundational reference for future model development based on larger datasets of patients treated for ALSS.

Secondly, although LSTM networks are widely used in clinical prediction due to their temporal modeling capabilities, their predictive performance in this study was significantly inferior to that of static models. This discrepancy primarily stems from the poor temporal resolution of the MIMIC dataset. Laboratory and vital sign data are sparsely sampled, irregular, and discontinuous—particularly for variables such as INR and fibrinogen—making it difficult to construct high-quality time series inputs. In addition, the short length of ICU stays in some patients resulted in insufficient sequence data, further limiting the model’s ability to learn time-dependent patterns. In contrast, the RF model, which is based on aggregated features, is less reliant on data continuity and demonstrated superior robustness and generalizability. Therefore, under the constraints of low-frequency and sparse ICU data, static models may offer greater practical value.

To address the limitations of LSTM performance, we attempted to incorporate attention mechanisms to enhance the model’s ability to capture critical time points. However, due to the short feature sequences and missing key records, no significant performance improvement over LSTM was observed. In the future, we plan to further evaluate the potential of architectures, such as transformer on higher-quality, multicenter time series data to optimize temporal modeling outcomes.

Furthermore, although the RF model performed well within the MIMIC-IV database, we fully acknowledge that external validation is essential for assessing its generalizability. Given that MIMIC is derived from a single US center and exhibits gender imbalances, the model’s applicability to other populations remains uncertain. The current lack of publicly available ALSS-specific, patient-level data limits the feasibility of immediate external validation. We plan to collaborate with regional multicenter hospitals, particularly in Asian populations, to conduct prospective validation studies and improve the model’s cross-population adaptability.

In this study, the definition of coagulation dysfunction labels was primarily based on established clinical diagnostic criteria and expert consensus. However, due to the absence of explicitly annotated bleeding events or other coagulation-related outcomes in the MIMIC database, we were unable to validate the correlation between the threshold and actual clinical risk. We acknowledge that future work should incorporate specific clinical outcomes (eg, gastrointestinal bleeding and hemorrhagic shock) to reassess the clinical significance of the risk threshold. Furthermore, considering the complex etiology of liver failure, adopting etiology-specific or dynamically adjusted prediction thresholds may better reflect real-world clinical scenarios and improve model sensitivity and practicality. We have identified this issue as a key direction for future research.

### Conclusion

In summary, this study not only provides systematic, evidence-based support for the efficacy of ALSS in improving coagulation function but also constructs a highly discriminative and interpretable risk prediction tool. The findings are of great significance for advancing individualized and intelligent coagulation support therapy and nursing interventions in patients with liver failure. Future efforts should aim to expand the model’s applicability by incorporating multicenter data and nursing practice variables, thereby promoting the implementation and refinement of dynamic coagulation monitoring systems and providing data-driven decision support in critical care settings.

Although the RF model demonstrated superior performance in predicting coagulation dysfunction in patients with liver failure, it is currently well-suited for practical use due to its clear structure and transparent feature inputs. Key predictors such as dynamic INR, PTT, and fibrinogen can be visualized through a graphical user interface, allowing clinicians and nursing staff to intuitively assess individual risk and determine optimal intervention timing. In the future, we plan to deploy this model as an open-access web application or integrate it into hospital information systems, enabling rapid clinical access to short-term coagulation risk predictions. This will support intelligent optimization of ALSS treatment pathways and nursing resource allocation. We believe this translational approach will significantly enhance the model’s clinical accessibility and practical value.

## Appendix

Multimedia Appendix 1 Machine learning for liver failure coagulation.

## Abbreviations

ACLF: acute-on-chronic liver failure
ALF: acute liver failure
ALS: artificial liver support
ALSS: artificial liver support system
AP: average precision
APTT: activated partial thromboplastin time
AUC: area under the curve
BIDMC: Beth Israel Deaconess Medical Center
BP: blood pressure
CRRT: continuous renal replacement therapy
HBV: hepatitis B virus
ICU: intensive care unit
INR: international normalized ratio
LR: logistic regression
LSTM: long short-term memory
MARS: molecular adsorbent recirculating system
MIMIC: Medical Information Mart for Intensive Care
MIT: Massachusetts Institute of Technology
NOS: Newcastle-Ottawa scale
PE: plasma exchange
PP: plasma perfusion
PR: precision-recall
PRISMA: Preferred Reporting Items for Systematic Reviews and Meta-Analyses
PT: prothrombin time
PTT: partial thromboplastin time
RF: random forest
ROC: receiver operating characteristic
SHAP: Shapley Additive Explanations
TBIL: total bilirubin
XGBoost: extreme gradient boosting

## Data Availability

All data generated or analyzed during this study are included in this article and/or its supplementary material files. Further enquiries can be directed to the corresponding author.
