# Supplementary material for: Coagulation Risk Prediction in Patients With Liver Failure: Integrated Meta-Analysis and Machine Learning Model Study
Source: JMIR Med Inform. 2025 Dec 8;13:e76348. doi: 10.2196/76348 (PMC12723362; doi:10.2196/76348)
Supplement: Multimedia Appendix 1 [file medinform_v13i1e76348_app1.docx]

**Table S1.** Detailed Search Strategies for Systematic Literature Retrieval Across Multiple Databases.

| **Search number** | **Query** | **Sort By** | **Filters** | **Search Details** | **Results** | **Time** |
| --- | --- | --- | --- | --- | --- | --- |
| 18 | (((Hepatic Failure[Title/Abstract]) OR ("Liver Failure"[Mesh])) AND (((((((((((((("Liver, Artificial"[Mesh]) OR (Artificial Liver[Title/Abstract])) OR (Artificial Livers[Title/Abstract])) OR (Livers, Artificial[Title/Abstract])) OR (Bioartificial Liver[Title/Abstract])) OR (Bioartificial Livers[Title/Abstract])) OR (Liver, Bioartificial[Title/Abstract])) OR (Livers, Bioartificial[Title/Abstract])) OR (Plasmapheresis[Title/Abstract])) OR (Double Plasma Exchange[Title/Abstract])) OR (Molecular Adsorbents Recirculating System[Title/Abstract])) OR (Selective Plasma Adsorption[Title/Abstract])) OR (Continuous Blood Purification[Title/Abstract])) OR (Cell-Based Bioartificial Liver[Title/Abstract]))) AND ((((((((((((("Blood Coagulation"[Mesh]) OR (Blood Clotting[Title/Abstract])) OR (Blood Clottings[Title/Abstract])) OR (Clotting, Blood[Title/Abstract])) OR (Coagulation, Blood[Title/Abstract])) OR (Coagulation indicators[Title/Abstract])) OR (Coagulation function[Title/Abstract])) OR (Coagulation Markers[Title/Abstract])) OR (Prothrombin Time[Title/Abstract])) OR (Activated Partial Thromboplastin Time[Title/Abstract])) OR (D-dimer[Title/Abstract])) OR (International Normalized Ratio[Title/Abstract])) OR (Fibrinogen[Title/Abstract])) | | | ("hepatic failure"[Title/Abstract] OR "Liver Failure"[MeSH Terms]) AND ("liver, artificial"[MeSH Terms] OR "artificial liver"[Title/Abstract] OR "artificial livers"[Title/Abstract] OR (("Liver"[MeSH Terms] OR "Liver"[All Fields] OR "Livers"[All Fields] OR "liver s"[All Fields]) AND "Artificial"[Title/Abstract]) OR "bioartificial liver"[Title/Abstract] OR "bioartificial livers"[Title/Abstract] OR (("Liver"[MeSH Terms] OR "Liver"[All Fields] OR "Livers"[All Fields] OR "liver s"[All Fields]) AND "Bioartificial"[Title/Abstract]) OR (("Liver"[MeSH Terms] OR "Liver"[All Fields] OR "Livers"[All Fields] OR "liver s"[All Fields]) AND "Bioartificial"[Title/Abstract]) OR "Plasmapheresis"[Title/Abstract] OR (("double"[All Fields] OR "doubled"[All Fields] OR "doubles"[All Fields] OR "doubling"[All Fields] OR "doublings"[All Fields]) AND "plasma exchange"[Title/Abstract]) OR "molecular adsorbents recirculating system"[Title/Abstract] OR "selective plasma adsorption"[Title/Abstract] OR "continuous blood purification"[Title/Abstract] OR ("Cell-Based"[All Fields] AND "bioartificial liver"[Title/Abstract])) AND ("Blood Coagulation"[MeSH Terms] OR "blood clotting"[Title/Abstract] OR (("Blood"[MeSH Subheading] OR "Blood"[All Fields] OR "Blood"[MeSH Terms] OR "bloods"[All Fields] OR "haematology"[All Fields] OR "hematology"[MeSH Terms] OR "hematology"[All Fields] OR "haematoma"[All Fields] OR "hematoma"[MeSH Terms] OR "hematoma"[All Fields] OR "haemorrhage"[All Fields] OR "hemorrhage"[MeSH Terms] OR "hemorrhage"[All Fields] OR "haemorrhages"[All Fields] OR "hemorrhages"[All Fields] OR "haemorrhagic"[All Fields] OR "haemorrhaging"[All Fields] OR "hematologies"[All Fields] OR "haematomas"[All Fields] OR "hematomas"[All Fields] OR "hematoma s"[All Fields] OR "hematomae"[All Fields] OR "hemorrhaged"[All Fields] OR "hemorrhagic"[All Fields] OR "hemorrhagical"[All Fields] OR "hemorrhaging"[All Fields]) AND "Clottings"[Title/Abstract]) OR "clotting blood"[Title/Abstract] OR "coagulation blood"[Title/Abstract] OR "coagulation indicators"[Title/Abstract] OR "coagulation function"[Title/Abstract] OR "coagulation markers"[Title/Abstract] OR "prothrombin time"[Title/Abstract] OR "activated partial thromboplastin time"[Title/Abstract] OR "D-dimer"[Title/Abstract] OR "international normalized ratio"[Title/Abstract] OR "Fibrinogen"[Title/Abstract]) | 112 | 3:07:19 |
| 17 | (((((((((((("Blood Coagulation"[Mesh]) OR (Blood Clotting[Title/Abstract])) OR (Blood Clottings[Title/Abstract])) OR (Clotting, Blood[Title/Abstract])) OR (Coagulation, Blood[Title/Abstract])) OR (Coagulation indicators[Title/Abstract])) OR (Coagulation function[Title/Abstract])) OR (Coagulation Markers[Title/Abstract])) OR (Prothrombin Time[Title/Abstract])) OR (Activated Partial Thromboplastin Time[Title/Abstract])) OR (D-dimer[Title/Abstract])) OR (International Normalized Ratio[Title/Abstract])) OR (Fibrinogen[Title/Abstract]) | | | "Blood Coagulation"[MeSH Terms] OR "blood clotting"[Title/Abstract] OR (("Blood"[MeSH Subheading] OR "Blood"[All Fields] OR "Blood"[MeSH Terms] OR "bloods"[All Fields] OR "haematology"[All Fields] OR "hematology"[MeSH Terms] OR "hematology"[All Fields] OR "haematoma"[All Fields] OR "hematoma"[MeSH Terms] OR "hematoma"[All Fields] OR "haemorrhage"[All Fields] OR "hemorrhage"[MeSH Terms] OR "hemorrhage"[All Fields] OR "haemorrhages"[All Fields] OR "hemorrhages"[All Fields] OR "haemorrhagic"[All Fields] OR "haemorrhaging"[All Fields] OR "hematologies"[All Fields] OR "haematomas"[All Fields] OR "hematomas"[All Fields] OR "hematoma s"[All Fields] OR "hematomae"[All Fields] OR "hemorrhaged"[All Fields] OR "hemorrhagic"[All Fields] OR "hemorrhagical"[All Fields] OR "hemorrhaging"[All Fields]) AND "Clottings"[Title/Abstract]) OR "clotting blood"[Title/Abstract] OR "coagulation blood"[Title/Abstract] OR "coagulation indicators"[Title/Abstract] OR "coagulation function"[Title/Abstract] OR "coagulation markers"[Title/Abstract] OR "prothrombin time"[Title/Abstract] OR "activated partial thromboplastin time"[Title/Abstract] OR "D-dimer"[Title/Abstract] OR "international normalized ratio"[Title/Abstract] OR "Fibrinogen"[Title/Abstract] | 148,220 | 3:04:44 |
| 16 | ((((((((((((("Liver, Artificial"[Mesh]) OR (Artificial Liver[Title/Abstract])) OR (Artificial Livers[Title/Abstract])) OR (Livers, Artificial[Title/Abstract])) OR (Bioartificial Liver[Title/Abstract])) OR (Bioartificial Livers[Title/Abstract])) OR (Liver, Bioartificial[Title/Abstract])) OR (Livers, Bioartificial[Title/Abstract])) OR (Plasmapheresis[Title/Abstract])) OR (Double Plasma Exchange[Title/Abstract])) OR (Molecular Adsorbents Recirculating System[Title/Abstract])) OR (Selective Plasma Adsorption[Title/Abstract])) OR (Continuous Blood Purification[Title/Abstract])) OR (Cell-Based Bioartificial Liver[Title/Abstract]) | | | "liver, artificial"[MeSH Terms] OR "artificial liver"[Title/Abstract] OR "artificial livers"[Title/Abstract] OR (("Liver"[MeSH Terms] OR "Liver"[All Fields] OR "Livers"[All Fields] OR "liver s"[All Fields]) AND "Artificial"[Title/Abstract]) OR "bioartificial liver"[Title/Abstract] OR "bioartificial livers"[Title/Abstract] OR (("Liver"[MeSH Terms] OR "Liver"[All Fields] OR "Livers"[All Fields] OR "liver s"[All Fields]) AND "Bioartificial"[Title/Abstract]) OR (("Liver"[MeSH Terms] OR "Liver"[All Fields] OR "Livers"[All Fields] OR "liver s"[All Fields]) AND "Bioartificial"[Title/Abstract]) OR "Plasmapheresis"[Title/Abstract] OR (("double"[All Fields] OR "doubled"[All Fields] OR "doubles"[All Fields] OR "doubling"[All Fields] OR "doublings"[All Fields]) AND "plasma exchange"[Title/Abstract]) OR "molecular adsorbents recirculating system"[Title/Abstract] OR "selective plasma adsorption"[Title/Abstract] OR "continuous blood purification"[Title/Abstract] OR ("Cell-Based"[All Fields] AND "bioartificial liver"[Title/Abstract]) | 19,066 | 2:58:42 |
| 12 | (Hepatic Failure[Title/Abstract]) OR ("Liver Failure"[Mesh]) | | | "hepatic failure"[Title/Abstract] OR "Liver Failure"[MeSH Terms] | 37,404 | 21:45:32 |

**Table S2.** Model Hyperparameters Used in This Study

| **A. Logistic Regression (Scikit-learn)** | |
| --- | --- |
| Parameter | Value |
| penalty | 'l2' |
| solver | 'lbfgs' |
| max_iter | 2000 |
| random_state | 42 |
| C | 1.0 |
| class_weight | None |
| **B. Random Forest (Scikit-learn)** | |
| Parameter | Value |
| n_estimators | 100 |
| criterion | 'gini' |
| max_depth | None |
| min_samples_split | 2 |
| random_state | 42 |
| **C. XGBoost Classifier** | |
| Parameter | Value |
| use_label_encoder | False |
| eval_metric | 'logloss' |
| random_state | 42 |
| n_estimators | 100 |
| max_depth | 6 |
| **D. LSTM Neural Network (TensorFlow)** | |
| Parameter | Value |
| Input shape | (5, feature_dim) |
| LSTM units | 64 |
| Dropout rate (1) | 0.3 |
| Dense units | 32 |
| Dropout rate (2) | 0.2 |
| Output activation | 'sigmoid' |
| Loss function | 'binary_crossentropy' |
| Optimizer | 'adam' |
| Epochs | 20 |
| Batch size | 64 |
| Validation split | 0.2 |
| EarlyStopping monitor | 'val_loss' |
| EarlyStopping patience | 3 |


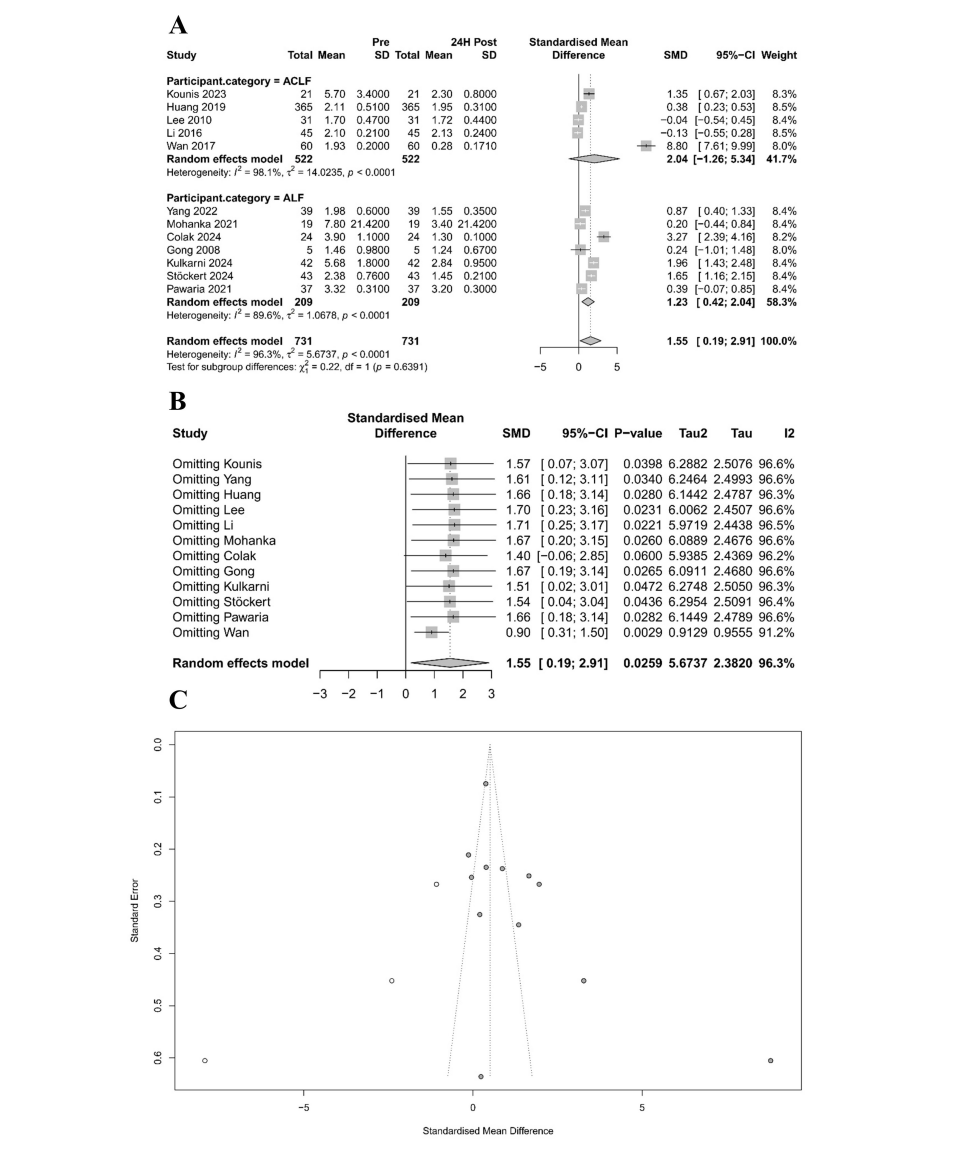


**Figure S1. Subgroup, Sensitivity, and Publication Bias Analyses of the Meta-Analysis on the Effect of ALS on INR Improvement.**

Note: (A) Forest plot of subgroup analysis stratified by disease type (ACLF vs. ALF); (B) Sensitivity analysis with sequential exclusion of individual studies to evaluate result robustness; (C) Funnel plot assessing publication bias. Abbreviations: SMD, standardized mean difference; 95% CI, 95% confidence interval.


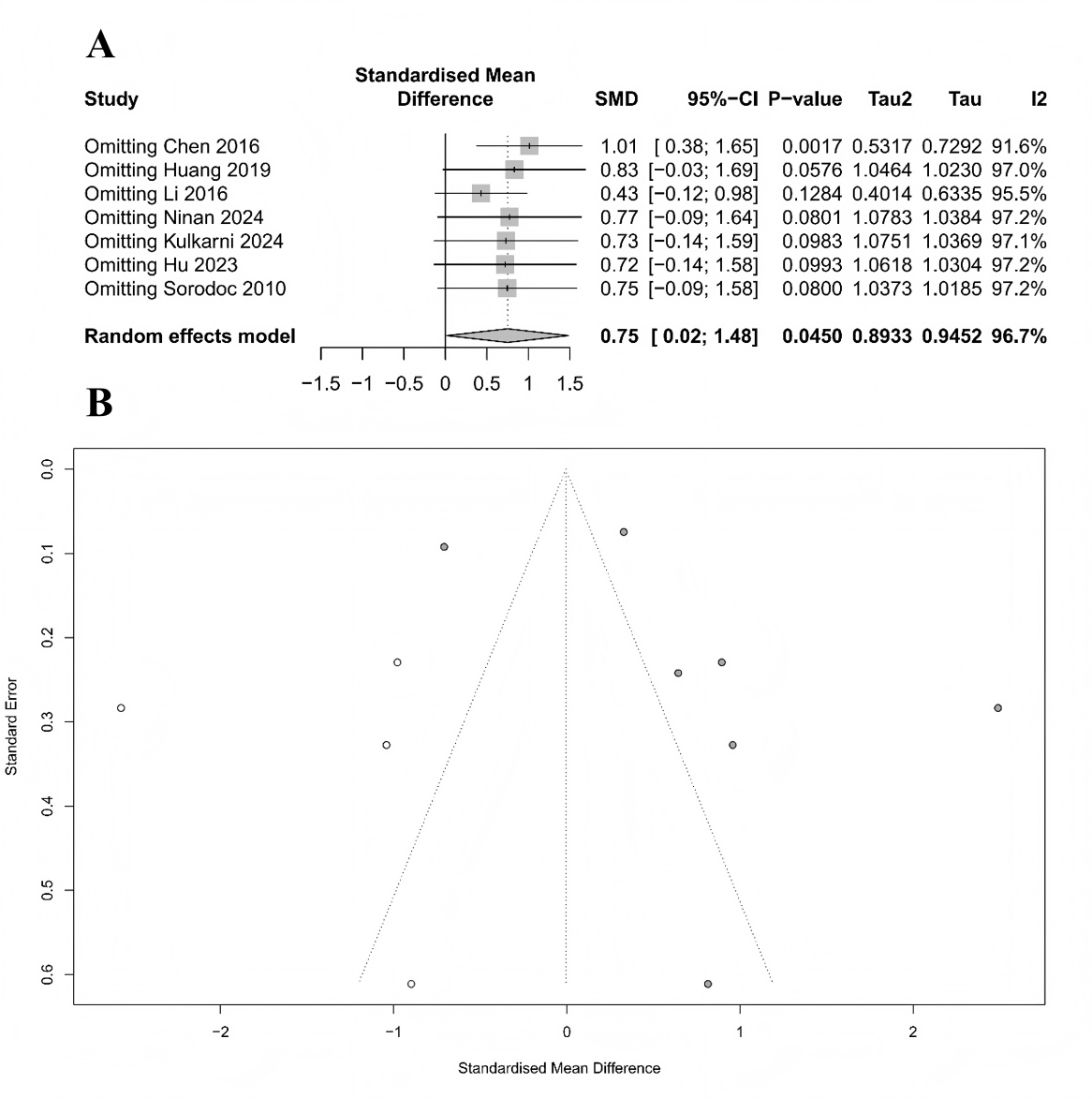


**Figure S2. Sensitivity Analysis and Publication Bias Assessment of PT Levels before and after ALS Therapy.**

Note: (A) Sensitivity analysis evaluating the influence of individual studies on the pooled effect size; (B) Funnel plot assessing publication bias among included studies. Abbreviations: SMD, standardized mean difference; 95% CI, 95% confidence interval.


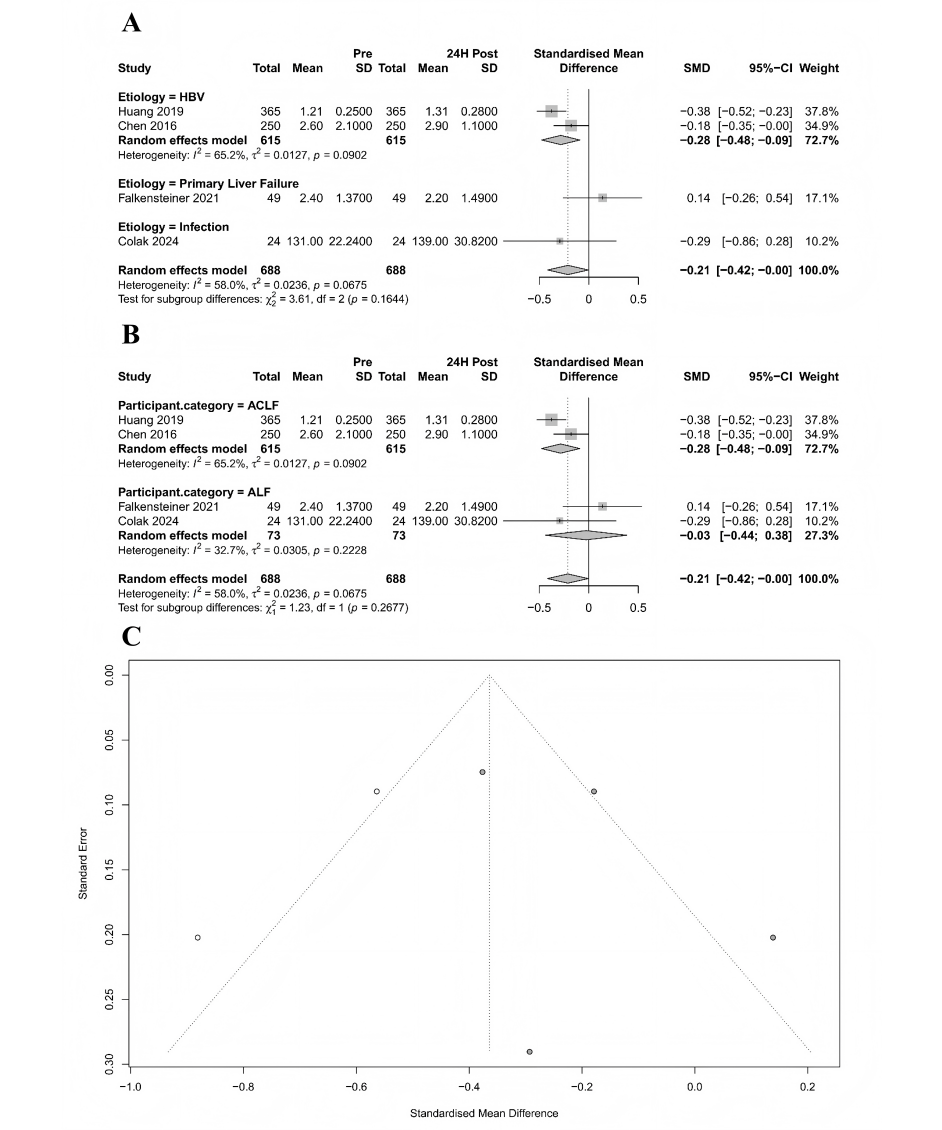


**Figure S3. Supplementary Meta-Analysis of the Effect of ALS on Fibrinogen Levels.**

Note: (A) Forest plot of fibrinogen level changes pre- and post-ALS stratified by etiology; (B) Forest plot of fibrinogen level changes pre- and post-ALS stratified by disease type; (C) Funnel plot assessing publication bias for fibrinogen-related studies. Abbreviations: SMD, standardized mean difference; 95% CI, 95% confidence interval.


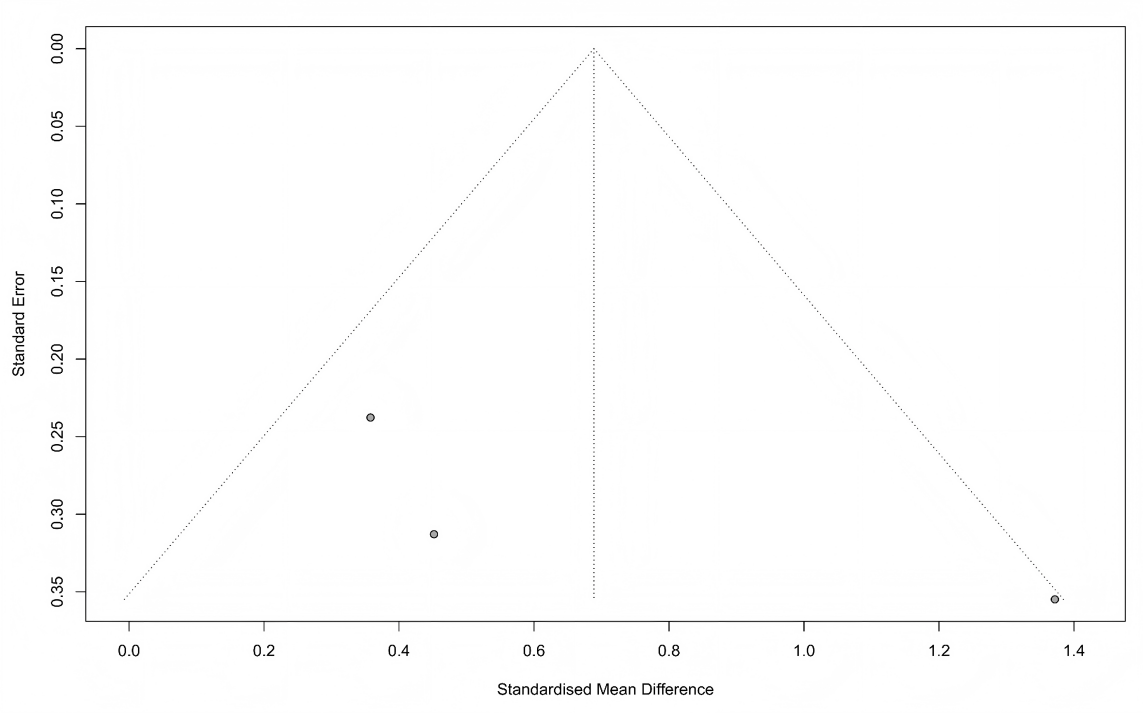


**Figure S4. Publication Bias Assessment for the Meta-Analysis of ALS Effects on APTT in Liver Failure Patients.**


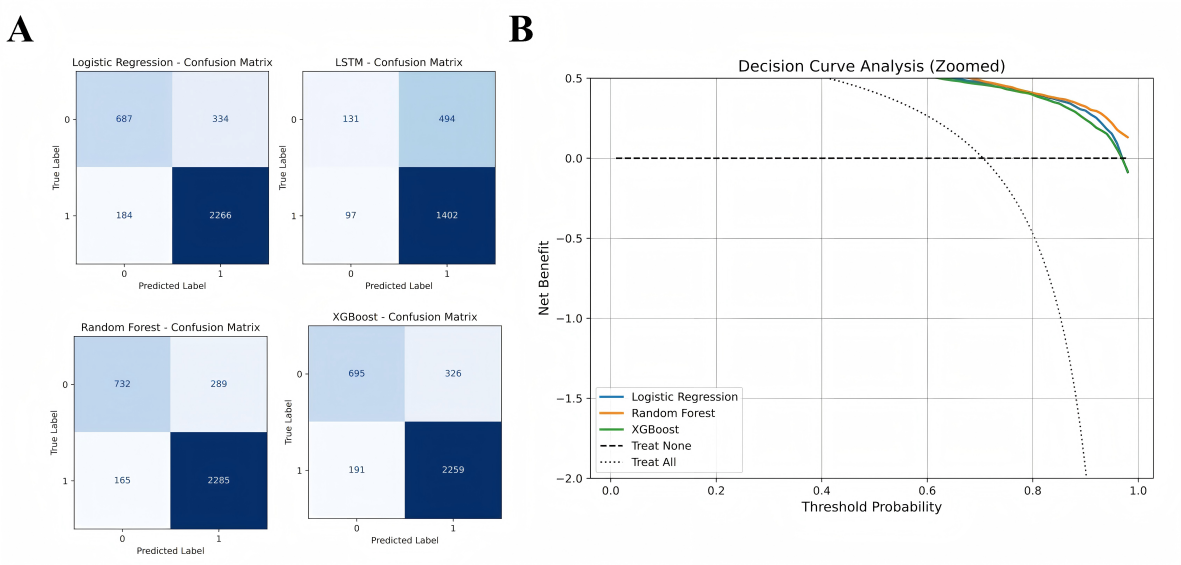


**Figure S5. Performance and Clinical Net Benefit Analysis of Machine Learning Models in Predicting Coagulation Dysfunction in Liver Failure Patients.**

Note: (A) Confusion matrices evaluating the classification accuracy of LR, RF, XGBoost, and LSTM models in distinguishing abnormal coagulation states within the independent test set; (B) DCA comparing the net clinical benefit of each model across threshold probabilities to assess their clinical decision-support utility.
